# Natural infection with *Leishmania* (*Mundinia*) *martiniquensis* supports *Culicoides peregrinus* (Diptera: Ceratopogonidae) as a potential vector of leishmaniasis and characterization of a *Crithidia* sp. isolated from the midges

**DOI:** 10.3389/fmicb.2023.1235254

**Published:** 2023-08-22

**Authors:** Saowalak Kaewmee, Chonlada Mano, Thanari Phanitchakun, Rinnara Ampol, Thippawan Yasanga, Urassaya Pattanawong, Anuluck Junkum, Padet Siriyasatien, Paul A. Bates, Narissara Jariyapan

**Affiliations:** ^1^Medical Parasitology Program, Department of Parasitology, Faculty of Medicine, Chulalongkorn University, Bangkok, Thailand; ^2^Center of Insect Vector Study, Department of Parasitology, Faculty of Medicine, Chiang Mai University, Chiang Mai, Thailand; ^3^Center of Excellence in Vector Biology and Vector-Borne Disease, Department of Parasitology, Faculty of Medicine, Chulalongkorn University, Bangkok, Thailand; ^4^Medical Science Research Equipment Center, Faculty of Medicine, Chiang Mai University, Chiang Mai, Thailand; ^5^Molecular Biology of Malaria and Opportunistic Parasites Research Unit, Department of Parasitology, Faculty of Medicine, Chulalongkorn University, Bangkok, Thailand; ^6^Division of Biomedical and Life Sciences, Faculty of Health and Medicine, Lancaster University, Lancaster, United Kingdom

**Keywords:** *Leishmania*, leishmaniasis, *Crithidia*, Trypanosomatids, *Leishmania martiniquensis*, *Culicoides*, *Culicoides peregrinus*, biting midges

## Abstract

The prevalence of autochthonous leishmaniasis in Thailand is increasing but the natural vectors that are responsible for transmission remain unknown. Experimental *in vivo* infections in *Culicoides* spp. *with Leishmania* (*Mundinia*) *martiniquensis* and *Leishmania* (*Mundinia*) *orientalis*, the major causative pathogens in Thailand, have demonstrated that biting midges can act as competent vectors. Therefore, the isolation and detection of *Leishmania* and other trypanosomatids were performed in biting midges collected at a field site in an endemic area of leishmaniasis in Tha Ruea and a mixed farm of chickens, goats, and cattle in Khuan Phang, Nakhon Si Thammarat province, southern Thailand. Results showed that *Culicoides peregrinus* was the abundant species (>84%) found in both locations and only cow blood DNA was detected in engorged females. Microscopic examination revealed various forms of *Leishmania* promastigotes in the foregut of several *C. peregrinus* in the absence of bloodmeal remnants, indicating established infections. Molecular identification using ITS1 and 3’UTR *HSP70* type I markers showed that the *Leishmania* parasites found in the midges were *L. martiniquensis*. The infection rate of *L. martiniquensis* in the collected flies was 2% in Tha Ruea and 6% in Khuan Phang, but no *L. orientalis* DNA or parasites were found. Additionally, organisms from two different clades of *Crithidia*, both possibly new species, were identified using SSU rRNA and gGAPDH genes. Choanomastigotes and promastigotes of both *Crithidia* spp. were observed in the hindgut of the dissected *C. peregrinus*. Interestingly, midges infected with both *L. martiniquensis* and *Crithidia* were found. Moreover, four strains of *Crithidia* from one of the clades were successfully isolated into culture. These parasites could grow at 37°C in the culture and infect BALB/c mice macrophages but no multiplication was observed, suggesting they are thermotolerant monoxenous trypanosomatids similar to *Cr. thermophila.* These findings provide the first evidence of natural infection of *L. martiniquensis* in *C. peregrinus* supporting it as a potential vector of *L. martiniquensis*.

## Introduction

Leishmaniases are vector-borne diseases caused by protozoan parasites of the genus *Leishmania* (Kinetoplastida, Trypanosomatidae). At least 21 *Leishmania* species that are members of the subgenera *Viannia*, *Leishmania*, and *Mundinia* have been reported as human pathogens. So far, no vaccine is available for human leishmaniasis (World Health Organization, 2023). In Thailand, human cases of autochthonous leishmaniases are mainly caused by two recently described species, *L*. (*Mundinia*) *martiniquensis* (Pothirat et al., 2014) and *L*. (*Mundinia*) *orientalis* (Jariyapan et al., 2018). The prevalence of autochthonous leishmaniasis in Thailand is increasing and new cases continue to be reported (Anugulruengkitt et al., 2022; Srivarasat et al., 2022), but the natural vectors that are responsible for the transmission of the disease remain unknown. Moreover, more relapse cases caused by *L. martiniquensis* have been reported (Srivarasat et al., 2022; Mano et al., 2023). Various species of sand flies are known as natural vectors of *Leishmania* parasites in the subgenera *Viannia* and *Leishmania* (Cecílio et al., 2022). For *L. martiniquensis* and *L. orientalis*, some species of sand flies have been reported as potential vectors (Chusri et al., 2014; Siripattanapipong et al., 2018; Srisuton et al., 2019; Sriwongpan et al., 2021). However, no natural vectors have been proved.

Recently, various research groups in several countries have reported the detection of *Leishmania* DNA in biting midges. For example, DNA of *L. infantum* has been detected in wild-caught *Culicoides* spp. in Tunisia (Slama et al., 2014), *L*. (*Viannia*) *braziliensis* DNA found in *C*. *ignacioi*, *C. insignis*, and *C. foxi* and *L*. (*Leishmania*) *amazonensis* DNA detected in *C*. *filariferus* and *C*. *flavivenula* in Brazil (Rebêlo et al., 2016). Further, in Australia, the natural infection of a day-feeding midge, subgenus *Forcipomyia* (*Lasiohelea*) Kieffer, with *L*. (*Mundinia*) *macropodum* parasites has been reported (Dougall et al., 2011). Experimental infections of *L*. (*Mundinia*) *enriettii* and *L. orientalis* in a laboratory colony of *C. sonorensis* reveals that both *Leishmania* species can complete their development to late-stage parasites found in the stomodeal valve, a position suitable for transmission by bite (Seblova et al., 2012; Chanmol et al., 2019). Further, Becvar and colleagues (Becvar et al., 2021) have demonstrated that *L. martiniquensis*, *L. orientalis*, and *Leishmania* (*Mundinia*) *chancei* (formerly called *L*. “Ghana” sp.) (Kwakye-Nuako et al., 2023), all members of subgenus *Mundinia*, are able to successfully colonize at the stomodeal valve, produce a higher proportion of metacyclic forms than in sand flies, and can be experimentally transmitted to BALB/c mice by *C. sonorensis* bites. These findings have highlighted *Culicoides* spp. as potential vectors of the members of the subgenus *Mundinia* that may participate in leishmaniasis transmission in nature (Becvar et al., 2021).

Biting midges are also probable vectors of avian trypanosomes. DNA of *Trypanosoma* spp. parasites has been detected in several wild caught *Culicoides* spp. (Bernotienė et al., 2020). *C*. *alazanicus*, *C. pictipennis*, *C*. *festivipennis*, and *C*. *clastrieri* are naturally infected with *T. bennetti* (s. l.) (Svobodová et al., 2017). Experimental infection of *C*. *nubeculosus* and *C. impunctatus* with four closely related haplotypes of *T. everetti* has shown that these *Trypanosoma* parasites are able to develop and produce metacyclic trypomastigotes in both biting midge species (Bernotienė et al., 2020).

Besides the dixenous trypanosomatids (*Leishmania* and *Trypanosoma*), natural infections of *Culicoides* biting midges with monoxenous trypanosomatids have been reported. *Herpetomonas ztiplika* was isolated from a female *C. kibunensis* caught while attacking buzzard (*Buteo buteo*) nestlings (Podlipaev S. A. et al., 2004). Several isolates of *Sergeia podlipaevi* were obtained from females of two species of biting midges *C*. *festivipennis* and *C. truncorum* captured in common buzzard nests (Svobodová et al., 2007). A monoxenous trypanosomatid, *Herpetomonas trimorpha*, was isolated from the digestive tract of a female biting midge, *C. truncorum* (Zídková et al., 2010). In addition, *Crithidia* sp. DNA was detected in one female *C. pictipennis* and *H*. *ztiplika* DNA in two *C. obsoletus* females (Bernotienė et al., 2020).

Infections with monoxenous trypanosomatids have occasionally been reported in mammals including humans. In mammals a flagellate parasite was isolated from rats (*Rattus norvegicus*) and stray dogs in Egypt (Morsy et al., 1988) and later found to be a member of the genus *Herpetomonas* (Podlipaev S. et al., 2004). Recently, mammals including coatis (*Nasua nasua*), marmosets (*Callithrix* sp.), bats (*Carollia perspicillata*, *Myotis lavali*, *M. izecksohni*, *Artibeus lituratus*), crab-eating foxes (*Cerdocyon thous*), and ocelots (*Leopardus pardalis*) in Brazil have been found infected in nature by *Crithidia mellificae*, a monoxenous trypanosomatid classically associated with honeybees (Dario et al., 2021). Also, DNA of *Cr*. *mellificae* is detected in a nectar-feeding bat (*Anoura caudifer*) in Brazil (Rangel et al., 2019).

So far, most infection by monoxenous trypanosomatids in humans has been reported in patients with either HIV or *Leishmania* co-infection. *Leptomonas seymouri*-*L. donovani* co-infection cases have been reported from India (Srivastava et al., 2010; Ghosh et al., 2012; Singh et al., 2013; Thakur et al., 2020). In Brazil, a *Crithidia-*related species has been isolated from an immunocompetent patient with a fatal visceral leishmaniasis-like illness (Maruyama et al., 2019) and a 9-year-old patient with leishmaniasis caused by *L. infantum* (Rogerio et al., 2023). Also, a novel thermotolerant monoxenous trypanosomatid closely related to *Cr*. *fasciculata* has been isolated from clinical samples of immunocompetent patients suspected of cutaneous leishmaniasis in Iran (Ghobakhloo et al., 2019; Kostygov et al., 2019). However, potential vectors of these monoxenous trypanosomatid parasites remain unknown.

In Thailand, the role of biting midges as potential vectors of *Leishmania* and *Trypanosoma* parasites has been investigated and revealed DNA of *L. martiniquensis* and *Trypanosoma* spp. in three female *C*. *mahasarakhamense* and one female *C*. *huffi*, respectively. Blood meal analysis showed *C. arakawae*, *C*. *mahasarakhamense*, *C*. *guttifer*, *C*. *huffi*, *C. fulvus* and *C*. *actoni* had fed on chickens, whereas *C*. *asiana*, *C*. *imicola*, *C. peregrinus*, *C*. *oxystoma* and *C*. *shortti* had fed on water buffalo and cattle (Jomkumsing et al., 2021; Sunantaraporn et al., 2021). Recently, DNA of *L. martiniquensis* was detected in *C. peregrinus*, *C. oxystoma*, *C. mahasarakhamense*, *C. huffi*, *C. fordae*, and *C. fulvus* and DNA of *L. orientalis* in *C. peregrinus* and *C. oxystoma* caught near a leishmaniasis patient’s house in southern Thailand. In addition, DNA of *Crithidia* sp. was detected in *Culicoides* spp. in those areas (Songumpai et al., 2022; Sunantaraporn et al., 2022).

Observation and isolation of live *L. martiniquensis* and *L. orientalis* parasites in natural infections of *Culicoides* biting midges is still required as it is one of the key criteria used to incriminate a natural vector of leishmaniasis. Therefore, the objectives of this study were (1) to investigate trypanosomatids in *Culicoides* biting midges collected from an endemic area of leishmaniasis and a mixed farm of chickens, goats, and cattle in southern Thailand, (2) to analyze blood meals of engorged midges, and (3) to characterize any trypanosomatids successfully isolated into culture. Our study provided evidence for *C. peregrinus* as a potential vector of *L. martiniquensis* and revealed a novel thermotolerant *Crithidia* trypanosomatid that could infect mouse cells *in vitro*.

## Materials and methods

### Ethic statements

The use of animals in this study was approved by the animal research ethics committee of Chulalongkorn University Animal Care and Use Protocol (CU-ACUP), Faculty of Medicine, Chulalongkorn University, Bangkok, Thailand (COA No. 023/2564 and COA No. 011/2564).

### Study location and insect trapping

Collection of biting midges was conducted in two locations, Location 1: Tambon Tha Ruea (TR), an endemic area of leishmaniasis (8°22′42.0”N 99°58′32.0″E) and Location 2: Tambon Khuan Phang (KP), a mixed farm of chickens, goats, and cattle (8°09′35.2”N 99°56′33.8″E), Nakhon Si Thammarat province, southern Thailand. In each location, wild biting midges were collected using five Center for Disease Control and Prevention (CDC) miniature light traps (25 W bulb) with ultraviolet (UV) light for 4 nights in December 2021. Traps were operated from 6.00 pm to 10.00 pm. The midges were kept in plastic boxes covered by two layers of insect nets with moisture papers on the top and transported to the Vector Biology and Vector Borne Disease Research Unit, Department of Parasitology, Faculty of Medicine, Chulalongkorn University.

### Morphological identification and investigation of naturally infected wild-caught biting midges

Live insects were placed on ice to immobilize before investigating under a binocular stereoscopic microscope (SZX10; Olympus, Tokyo, Japan). Males were separated from females by their morphology. Species identification of females was carried out using a taxonomic key according to morphological characters, namely wing spot patterns and head features (palp and antenna) (Dyce et al., 2007; Pramual et al., 2021). In each species, females were grouped by their physiological stage: parous (empty abdomen, with traces of burgundy pigment after blood sucking), engorged (abdomen filled with blood), gravid (abdomen with eggs), and nulliparous (empty abdomen without the presence of blood, they never sucked blood) (Dyce, 1996; Kasičová et al., 2021). Parous (50) and nulliparous (50) females collected from each location were selected to investigate for trypanosomatids. Before dissection, the midges were placed on ice in a small Petri dish containing 0.05% (v/v) Tween 20 in 1× Phosphate Buffered Saline (PBS; 10 mM sodium phosphate, 145 mM sodium chloride, pH 7.2). The whole gut (foregut, midgut, and hindgut) was dissected using sterile fine needles in a drop of 50 μl sterile PBS on a sterile slide under a binocular stereoscopic microscope. Then the gut was transferred into 50 μl sterile PBS on a new sterile slide, covered with a sterile coverslip, and examined for trypanosomatids under a light microscope (Olympus America Inc., USA) at 400 × magnification. Some insects with unidentified trypanosomatids in the whole gut were video recorded and photographed. Each positive sample was divided into three parts in a small volume of PBS for Giemsa’s staining, cultivation, and gDNA extraction. To confirm the species of the infected insects, the female carcass of each positive sample was subjected to gDNA extraction for molecular identification of *Culicoides* species.

### Cultivation of trypanosomatids from insects

Schneider’s Insect medium (SIM) (Sigma-Aldrich, St Louis, MO, USA), pH 6.8 supplemented with 10% (v/v) heat-inactivated fetal bovine serum (hi-FBS) (Life Technologies-Gibco, Grand Island, NY, USA), 100 μg/mL penicillin–streptomycin and 250 μg/mL gentamicin (Sigma-Aldrich, St. Louis, MO, USA) was used to culture trypanosomatids from insects. Each positive sample in PBS was transferred into a 25 ml flask containing 5 mL SIM, pH 6.8 supplemented with 10% (v/v) hi-FBS and 25 μg/ml gentamicin (SIM complete), and incubated at 26°C. An axenic culture was established by serial dilution and subpassage. Promastigotes were mixed with 7.5% (v/v) glycerol in SIM complete and stored at −80°C.

### Giemsa staining, light microscopy and morphometry

Trypanosomatid samples isolated directly from insects or cultures in SIM with supplements were smeared on microscope slides and air-dried. The slides were then fixed in absolute methanol and stained with 5% (v/v) Giemsa’s stain solution (Sigma-Aldrich, Darmstadt, Germany). The morphological characteristics and morphometry of the trypanosomatids were examined under a light microscope (Olympus America Inc., USA) at 1,000 × magnification. Light microscopy (LM) images of 50 parasites in each stage were used for morphometry, including: cell body length, cell body width, anterior end to kinetoplast distance, anterior end to nucleus distance, and length of flagellum. The measurement results were presented as mean ± standard deviation.

### Genomic DNA extraction, molecular identification of *Culicoides* species, host blood, *Leishmania* species, and trypanosomatids

Genomic DNA of biting midges and trypanosomatids was extracted using a genomic DNA purification kit (Thermo Fisher Scientific Inc., Waltham, MA, USA) according to the manufacturer’s instructions. For molecular identification of *Culicoides* species, LCO1490 primer (5 ´–GGTCAACAAATCATAAAGATATTGG−3 ´) and HCO2198 primer (5 ´–TAAACTTCAGGGTGACCAAAAAA TCA−3 ´) (Folmer et al., 1994) were used to amplify the approximately 658-bp fragment of the mitochondrial cytochrome c oxidase subunit 1 gene (*COI*) following the polymerase chain reaction (PCR) method described by Harrup et al. (2016). Host blood from engorged females was identified based on the mitochondrial cytochrome b (*cyt b*) gene sequence using the primers cyt bb1 (5 ´–CCATCMAACATYTCADC ATGAAA−3 ´) and cyt bb2 (5 ´–GCHCCTCAGAATGAYATTTG KCCTCA−3 ´) as described by Radrova et al. (2013). For molecular identification of *Leishmania* species in gDNA samples extracted from insects and/or cultures, primers LeF (5 ´–TCCGCCCGAAAGTTCACC GATA−3 ´) and LeR (5 ´–CCAAGTCATCCATCGCGACACG−3 ´) (Spanakos et al., 2008), were used to amplify the internal transcript spacer 1 (ITS1) region (approximately 379 bp) and primers, 70-IR-D (5 ´-CCAAGGTCGAGGAGGTCGACTA-3 ´) and 70-IR-M (5 ´-ACG GGTAGGGGGAGGAAAGA −3 ´) (Requena et al., 2012) were used to amplify the 3 ´untranslated region (3 ´UTR) of *Leishmania HSP70*-type I (*HSP70-I*) genes as described by Jariyapan et al. (2021).

For molecular identification of trypanosomatids, primers, TRY927F (5 ´–GAAACAAGAAACACGGGAG −3 ´) and TRY927R (5 ´–CTACTGGGCAGCTTGGA−3 ´), were used to amplify approximately 927 bp of the small subunit ribosomal RNA (SSU rRNA) gene (Noyes et al., 1999) of gDNA extracted from insects and cultures. All amplicons were visualized in 1.5% agarose. The PCR products were purified using a GeneJET PCR Purification kit (Thermo Fisher Scientific, CA, USA). PCR conditions and protocols used in this study are provided in Supplementary Data S1.

For trypanosomatids successfully grown in culture, primers M200 (5 ´–ATGGCTCCVVTCAARGTWGGMAT−3 ´) and M201 (5 ´–TAKCCCCACTCRTTRTCRTACCA −3 ´) for the glycosomal glyceraldehyde-3-phosphate dehydrogenase (gGAPDH) gene (Maslov et al., 1996) were used to confirm species identification. The PCR products of M200/M201 primers were cloned into the pGEM-T Easy Vector system (Promega, Madison, WI, USA), following the manufacturer’s instructions. Recombinant plasmid DNA was extracted using the Invisorb Spin Plasmid Mini Kit (STRATEC Molecular, Berlin, Germany), following the manufacturer’s instructions. The purified PCR products were sent for sequencing at the sequencing service of Macrogen Inc., Seoul, Korea.

### Data and phylogenetic analyses

The nucleotide sequences of the *COI* gene of *Culicoides* biting midges, the *cyt b* gene of vertebrates, ITS1 and 3’UTR-*HSP70*-I genes of *Leishmania* parasites, and SSU rRNA and gGAPDH genes of trypanosomatids were analyzed by comparison with the GenBank database using a BLAST search^1^. The sequences of *Leishmania* parasites and trypanosomatids were aligned and trimmed using MEGA X: Molecular Evolutionary Genetics Analysis across computing platforms version 10.2.6 (Kumar et al., 2018). The best-fitting nucleotide substitution models for both genes were estimated using the Akaike Information Criterion (AIC) in the jModelTest software (Posada, 2008). Phylogenetic trees were constructed using the Maximum Likelihood (ML) method implemented in MEGA (Kumar et al., 2018) with 1,000 bootstrap-replications. Bootstrap values of ≥70% were taken as an indication support (Hillis and Bull, 1993). For phylogenetic analysis of *Leishmania* parasites, evolutionary models of GTR + I were chosen for ITS1 (329 bp) and 3’UTR-*HSP70*-I (817 bp). For phylogenetic analysis of *Crithidia* sp., evolutionary models of GTR + I + G were chosen for SSU rRNA (720 bp) and gGAPDH (654 bp). Genetic distances were calculated using the Kimura 2-parameter (K2P) model implemented in MEGA (Kumar et al., 2018).

### Scanning electron microscopy and transmission electron microscopy

Scanning electron microscopy (SEM) and transmission electron microscopy (TEM) were used to characterize the ultrastructural morphology of the cultured trypanosomatids. For SEM, live trypanosomatids were pelleted at 1,600 × *g* for 10 min at room temperature and fixed with 2.5% (v/v) glutaraldehyde in 0.1 cacodylate buffer (pH 7.2) overnight at 4°C. Fixed cells were washed twice in PBS and post-fixed with 1% (w/v) osmium tetroxide in PBS for 1 h. The samples were then dehydrated in a graded series of ethanol. Samples were critical point dried in liquid CO_2_ and coated with gold particles in a sputter-coating apparatus. Samples were observed under a scanning electron microscope, JEOL JSM-6610LV (JEOL, Tokyo, Japan), operated at 15 kV. Samples for TEM were prepared as for SEM, except that 2% (w/v) osmium tetroxide were used to post-fix for 2 h at room temperature. After dehydration in a graded series of ethanol, the samples were incubated overnight in an epoxy resin (PolyBed 812)/acetone solution (1:1), and then embedded in pure resin and polymerized for 48 h at 60°*C. ultra*-thin sections were stained with 4% (w/v) uranyl acetate and 3% (w/v) lead citrate and observed under a transmission electron microscope, JEM-2200FS (JEOL, Tokyo, Japan), operated at 200 kV.

### Growth curves at 26 and 37°C

Trypanosomatids were removed from −80°C, grown in SIM complete at 26°C for 4 days, and subpassaged into new SIM complete for growth analysis. The parasites were counted using a Neubauer chamber (BLAUBRAND, Sigma-Aldrich, Saint Louis, MO, USA) from an initial inoculum of 4 × 10^4^ parasites/mL (day 0). The initial inoculum was transferred into a 25 cm^3^ flask containing 5 ml of the culture medium for 14 flasks. Seven flasks were incubated at 26°C and the rest were incubated at 37°C with 5% CO_2_. One flask at each temperature was taken out daily, and a cell scraper (SPL Life Sciences, Gyeonggi, Korea) was applied to remove any adherent cells from the base of the flask. Ten microliters of parasites were collected and mixed at a 1:1 ratio with formaldehyde when all parasites were alive or 0.4% trypan blue solution (Thermo Fisher Scientific, NY, USA) when nonviable forms of parasites were present in the cultures. Live cells were counted using a Neubauer chamber. Also, the cultures were smeared on slides and stained with Giemsa’s staining solution for morphological examination by LM. Experiments were performed on three independent replicates run in duplicate.

### Macrophage infection and multiplication assay

To evaluate the ability of trypanosomatids to infect and multiply in mouse macrophages an infection and multiplication assay was performed. Mouse peritoneal exudate macrophages (PEMs) from BALB/c (*Mus musculus*) (Nomura Siam International Co., Ltd., Bangkok, Thailand) were obtained using the method described previously (Zhang et al., 2008). Round coverslips were placed in 24-well tissue culture plates (ThermoFisher Scientific, Jiangsu, China). PEMs in RPMI 1640 medium (GE Healthcare Life Science-HyClone, UT, United States) supplemented with gentamicin 25 μg/ml and 10% (v/v) hi-FBS were seeded at a density of 2.5 × 10^5^ cells/well and incubated at 37°C with 5% CO_2_ for 24 h. After the incubation, non-adherent cells were removed by washing three times with pre-warmed serum-free RPMI 1640 medium. Test trypanosomatids (from Day 3 with the greatest number of motile forms) and *L. martiniquensis* promastigotes (from Day 5) were used to infect PEMs at a ratio of 10:1 cells to macrophages. After incubation for 3 h at 37°C and 5% CO_2_, the coverslips were washed with pre-warmed serum-free RPMI 1640 medium for three times, replaced with 10% hi-FBS RPMI-1640 medium, and incubated at the same conditions. Coverslips were removed from the culture plates and stained with Giemsa’s staining solution every 24 h until 96 h post-infection. Two hundred macrophages were counted to determine the infection rate and average number of intracellular parasites per macrophage. The infection index, and the intracellular parasite multiplication ratio were determined (Chanmol et al., 2019). Results were expressed as mean ± standard deviation and based on three independent infection experiments each performed in duplicate.

### Statistical analysis

All statistical analyses were performed using GraphPad Prism version 9.1 software. Statistical differences between *L. martiniquensis* and *Crithidia* sp. infections at the different time points within one group were determined using two-way ANOVA with Bonferroni’s *post hoc* multiple comparisons for growth, infection index, and intracellular multiplication ratio. Tests were considered statistically significant if *p* < 0.05.

## Results

### *Culicoides* species presence, blood host, and *Leishmania* and trypanosomatids in *Culicoides peregrinus*

A total of 1,094 biting midges of the genus *Culicoides* were captured in 2 locations, 349 females (parous = 276, engorged = 2, nulliparous = 71) and 6 males from Tha Ruea and 724 females (parous = 527, engorged = 12, nulliparous =185) and 15 males from Khuan Phang (Supplementary Table S1). No gravid females were found in either location. Three species of biting midges were identified in Tha Ruea, *C. peregrinus* (*n* = 343, 96.62%), *C. mahasarakhamense* (*n* = 9, 2.53%), and *C. oxystoma* (*n* = 3, 0.85%). In Khuan Phang seven species were identified, *C. peregrinus* (*n* = 623, 84.3%), *C. imicola* (*n* = 31, 4.19%), *C. shortti* (*n* = 27, 3.65%), *C. huffi* (*n* = 25, 3.38%), *C. mahasarakhamense* (*n* = 20, 2.7%), *C. palpifer* (*n* = 11, 1.5%), and *C. oxystoma* (*n* = 2, 0.27%). Thus, in both locations *C. peregrinus* was the most abundant species. For engorged females (*n* = 14), all blood host identifications were of *Bos indicus* cattle (GenBank accession no. OR088254–OR088267).

**Figure 1:**
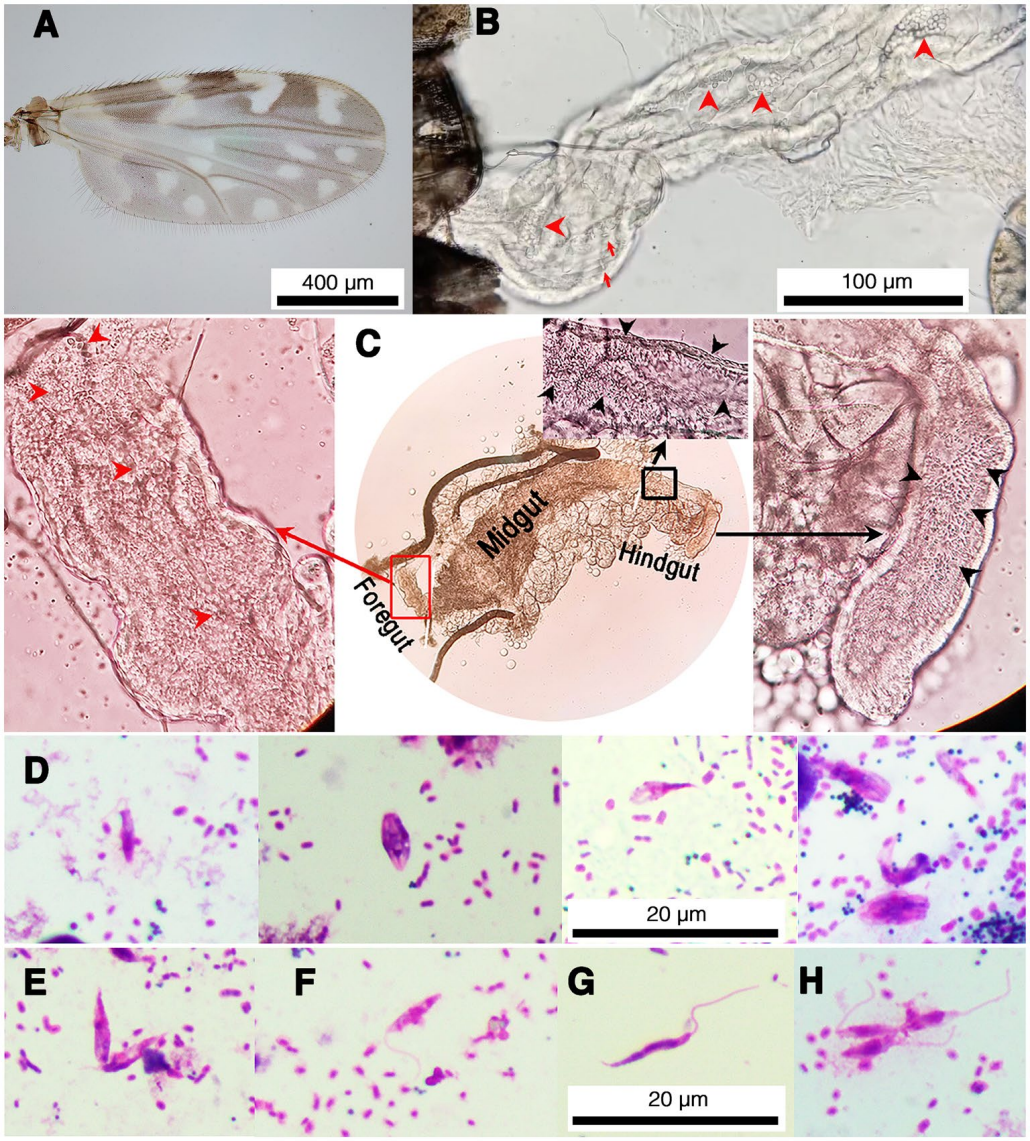
Representative images of trypanosomatids and co-infection of *L. martiniquensis* and *Crithidia* sp. in naturally infected *C. peregrinus* biting midges. **(A)** Wing pattern of *C. peregrinus*. **(B)** Trypanosomatids in the hindgut of the midge KP22. Red arrowheads indicate choanomastigotes and arrows indicate promastigotes. **(C)** *Leishmania* parasites and *Crithidia* sp. co-infected in the midge KP10. Red arrowheads and black arrowheads indicate trypanosomatids in the foregut and the hindgut, respectively. Red rectangle indicates an area in the foregut showing the movement of *Leishmania* parasites in Supplementary Video S1. Black rectangle indicates an area in the hindgut showing the movement of *Crithidia* sp. in Supplementary Video S2. **(D)** Representative of various forms of *Crithidia* sp. in the midge KP22. **(E–H)** Representative promastigote forms of *Leishmania* parasites in the midge KP19. **(E)** Procyclic promastigotes, aggregated form. **(F)** Procyclic promastigote. **(G)** Nectomonad promastigote. **(H)** Leptomonad promastigotes.

Fifty parous and 50 nulliparous females collected from each location were dissected for trypanosomatid infection. Trypanosomatids were observed under light microscopy (LM) in 26 out of the 100 samples of parous females from the two locations. However, no trypanosomatids were found in any nulliparous flies. All infected flies were identified as *C. peregrinus* by wing spot patterns (Figure 1A) and molecular methods (GenBank accession no. OR077408–OR077433). Light microscopic examination showed trypanosomatids present in the digestive tract of the midges (Figure 1 and Supplementary Videos S1, S2). None of the midges dissected had remains of bloodmeals in their midguts. Although the midges were dissected carefully, due to their small size and delicate nature, the digestive tracts of some midges were torn and trypanosomatids released. However, in most samples with an intact digestive tract trypanosomatids were found in the midgut and hindgut, with choanomastigote and/or promastigote morphologies observed (Figure 1B). Interestingly, trypanosomatids were observed in the foregut, midgut, and hindgut of one *C. peregrinus*, KP10 (Figure 1C). In this insect, video records revealed typical movement of *Leishmania* promastigotes in the foregut (Supplementary Video S1) and *Crithidia* in the hindgut (Supplementary Video S2). Molecular identification (see below) of the KP10 sample revealed that this midge was co-infected with *L. martiniquensis* and a possible novel *Crithidia* species (Table 1). Trypanosomatids were examined in slides prepared from infected midges, and various forms of promastigotes and chroanomastigotes were observed (Figure 1D). In the insect KP19 many promastigote forms of *Leishmania* were observed including procyclic promastigotes (Figures 1E,F), nectomonad promastigotes (Figure 1G), and leptomonad promastigotes (Figure 1H), however, no metacyclic promastigotes were observed. The parasites in KP19 were identified as *L. martiniquensis* by molecular methods (Table 1). The presence of *Leishmania* promastigotes in the foregut of midges TR17, KP10, KP17 and KP19 in the absence of bloodmeals are indicative of established infections.

**Table 1:** Natural infection of trypanosomatids in *C. peregrinus* collected from both locations.

Location: Tha Ruea		Matched species (Genbank accession no., % best match); [Genbank accession no. of the sequence in this study]			
Insect code	culture	Molecular targets			
		ITS1	3’UTR-*HSP70*-I	SSU rRNA^e^	gGAPDH
TR1, TR42	(−)	NM^a^	ND^b^	*Crithidia* sp. (OP698038, 99.46%); *Cr. thermophila* (KY264937.1, 97.64%); [OR077444- OR077445]	ND
TR2	(+)	NM	ND	*Crithidia* sp. (OP698038, 99.46%); *Cr. thermophila* (KY264937.1, 97.63%); [OR077446]	*Cr. fasciculata* (AF047493.1, 94.41%); [OR088272]
TR3	(+)	NM	ND	*Crithidia* sp. (OP698038, 99.46%); *Cr. thermophila* (KY264937.1, 97.64%); [OR077447]	*Cr. fasciculata* (AF047493.1, 94.60%); [OR088273]
TR17^c^	(−)	*L. martiniquensis* (MK603827.1, 98.94%); [OR077858]	*L. martiniquensis* (MK607437.1, 100%); [OR088268]	*Crithidia* sp. (OP698038, 99.46%); *Cr. thermophila* (KY264937.1, 97.64%); [OR077448]	ND
TR18	(−)	NM	ND	*Crithidia* sp. (OP698038, 98.92%); *Cr. thermophila* (KY264937.1, 97.31%), [OR077449]	ND
TR19	(−)	NM	ND	*Crithidia* sp. (OP698037, 99.24%); *Cr. thermophila* (KY264937.1, 97.00%); [OR077450]	ND
TR20, TR27, TR29	(−)	NM	ND	*Crithidia* sp. (OP217136, 100%); *Crithidia* sp. (MW694347.1, 99.03%); [OR077451 - OR077453]	ND
TR21	(−)	NM	ND	*Crithidia* sp. (OP217136, 99.89%); *Crithidia* sp. (MW694347.1, 98.92%); [OR077454]	ND
Location: Khuan Phang					
KP1	(+)	NM	ND	*Crithidia* sp. (OP698038, 99.57%); *Cr. thermophila* (KY264937.1, 97.64%); [OR077455]	*Cr. brachyflagelli* (JF717835.1, 94.61%); [OR088274]
KP2	(−)	NM	ND	*Crithidia* sp. (OP698038, 99.24%); *Cr. thermophila* (KY264937.1, 97.42%); [OR077456]	ND
KP3	(−)	NM	ND	*Crithidia* sp. (OP698037, 99.74%); *Cr. thermophila* (KY264937.1, 97.31%); [OR077457]	ND
KP4	(+)	NM	ND	*Crithidia* sp. (OP698038, 99.89%); *Cr. thermophila* (KY264937.1, 97.53%); [OR077458]	*Cr. fasciculata* (AF047493.1, 94.39%); [OR088275]
KP10^c^	(−)	*L. martiniquensis* (MK603827.1, 98.94%); [OR077859]	*L. martiniquensis* (MK607435.1, 98.64%); [OR088269]	*Crithidia* sp. (OP698038, 99.68%); *Cr. thermophila* (KY264937.1, 97.74%), [OR077459]	ND
KP17^d^	(−)	*L. martiniquensis* (MK603827.1, 98.94%); [OR077860]	*L. martiniquensis* (MK607435.1, 99.77%); [OR088270]	No PCR product	ND
KP18, KP28	(−)	NM	ND	*Crithidia* sp. (OP698038, 99.68%); *Cr. thermophila* (KY264937.1, 97.74%); [OR077460 - OR077461]	ND
KP19^d^	(−)	*L. martiniquensis* (MK603827.1, 98.94%); [OR077861]	*L. martiniquensis* (MK607437.1, 100%); [OR088271]	No PCR product	ND
KP22	(−)	NM	ND	*Crithidia* sp. (OP698037, 99.35%); *Cr. thermophila* (KY264937.1, 97.10%); [OR077462]	ND
KP24, KP25, KP30, KP32	(−)	NM	ND	*Crithidia* sp. (OP698037, 99.78%); *Cr. thermophila* (KY264937.1, 97.31%); [OR077463 - OR077466]	ND
KP47	(−)	NM	ND	*Crithidia* sp. (OP217136, 99.89%); *Crithidia* sp. (MW694347.1, 99.14%); [OR077467]	ND

^a^NM, not matched; ^b^ND, not done; ^c^Co-infection of *L. martiniquensis* and *Crithidia* sp.; ^d^*L. martiniquensis* infection; ^e^The best matched species and the relevant matched species.

Four isolates of trypanosomatids were successfully isolated into culture, two from *C. peregrinus* collected in Tha Ruea (TR2 and TR3) and two from *C. peregrinus* collected in Khuan Phang (KP1 and KP4) (Table 1). Unfortunately, other cultures including all those from midges with *L. martiniquensis* were too contaminated with bacteria and fungi and had to be discarded.

**Figure 2:**
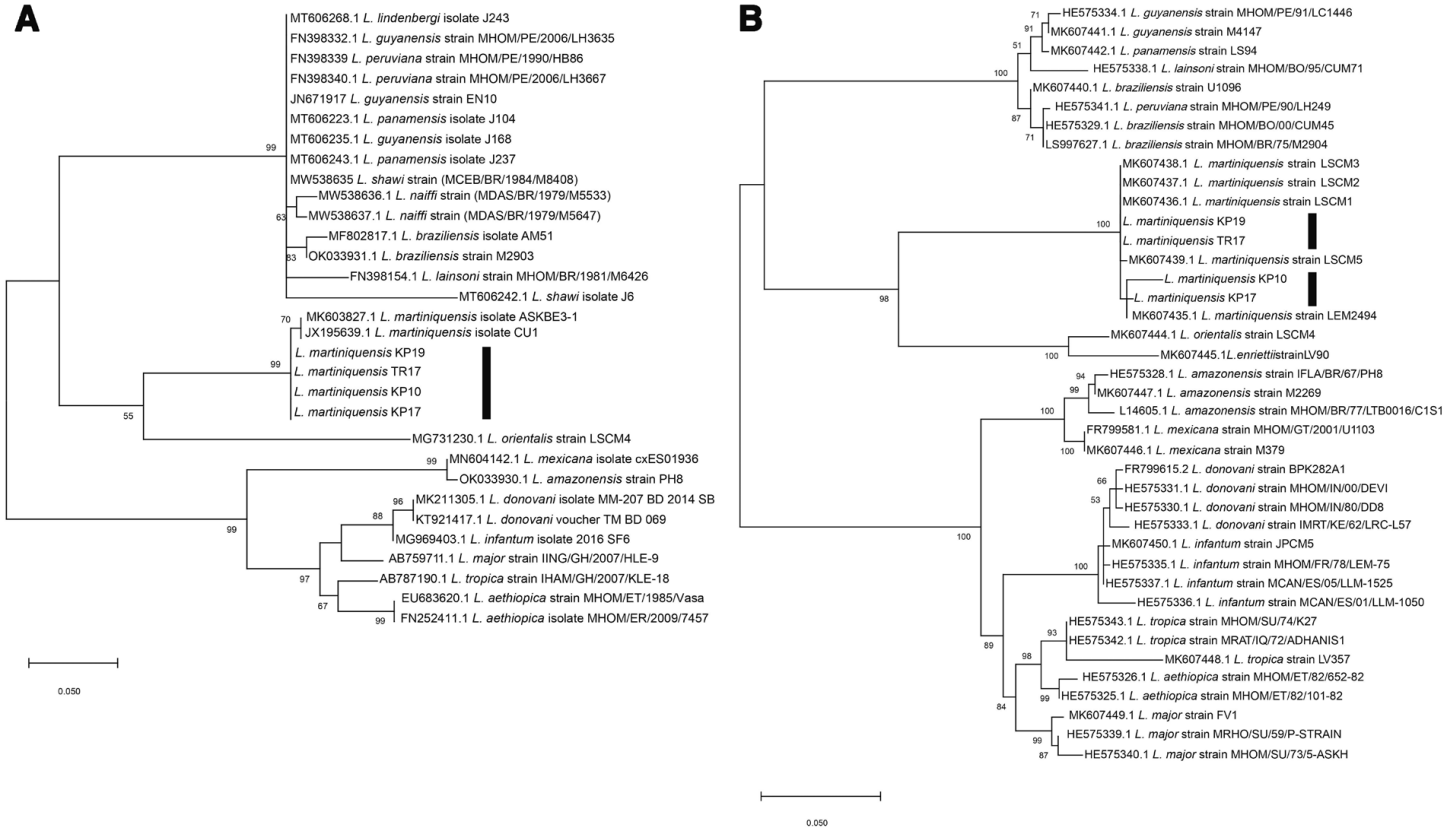
**(A)** Maximum Likelihood tree based on ITS1 sequences of *Leishmania* spp. **(B)** Maximum Likelihood tree based on 3’UTR-*HSP70*-I sequences of *Leishmania* spp. Bootstrap values are shown at each node. Bootstrap values of ≥70% correspond to supported clades. Nodes with Bootstrap values of <50% are not shown.

Molecular methods were used to identify the trypanosomatids. For molecular identification of *Leishmania* species, 100 gDNA samples extracted from the dissected insects were subjected to the PCR amplification of the ITS1 and 3’UTR-*HSP70*-I regions. Four samples of the dissected insects, from TR17, KP10, KP17, and KP19, were positive for *Leishmania* and their ITS1 amplicons were successfully sequenced. Moreover, a second molecular marker, 3’UTR-*HSP70*-I, was used to confirm the identification of the *Leishmania* species of the samples. BLAST analysis of the ITS1 and 3’UTR-*HSP70*-I sequences of the samples revealed that the ITS1 sequences were closest to that of *L. martiniquensis*, GenBank accession number MK603827.1, with 98.94% identity, whereas the 3’UTR-*HSP70*-I sequences were very similar (98.64 and 99.77% identity) or identical (100% identity) to that of *L. martiniquensis*, GenBank accession number MK607435.1, and another one was identical to *L. martiniquensis*, GenBank accession number MK607437.1 (Table 1). The ITS1 and 3’UTR-*HSP70*-I sequences were subjected to phylogenetic analyses together with other human *Leishmania* strains (Figure 2). The results from Maximum Likelihood tree based on amplified section of the ITS1 and 3’UTR-*HSP70*-I sequences showed that the new sequences fell in one clade with *L. martiniquensis* with 99% bootstrap values (K2P 0–0.78%) for ITS1 (Figure 2A) and 100% bootstrap values (K2P 0–1.63%) for 3’UTR-*HSP70*-I (Figure 2B). These results indicate that the trypanosomatid DNAs detected in the *C. peregrinus* samples, TR17, KP10, KP17, and KP19, were all of *L. martiniquensis*.

The SSU rRNA of 24 samples were successfully amplified and sequenced allowing the identification of other trypanosomatids. BLAST analysis showed that among these, 19 sequences were similar to each other and to a *Crithidia* sp. reported from Thailand by Songumpai et al. (2022) (GenBank accession number OP698037–OP698038) with 99.35–100% identity and to *Cr. thermophila* (GenBank accession number KY264937.1) with 97.00–97.74% identity. The other five sequences were also very similar to another *Crithidia* sp. reported from Thailand by Songumpai et al. (2022) (GenBank accession number OP217136) and Sunantaraporn et al. (2022) (GenBank accession number MW694347.1) with 99.89–100% and 98.92–99.14% identity, respectively (Table 1). These data showed that two midges, TR17 and KP10, were co-infected with both *L. martinquensis* and *Crithidia*, as suspected from dissection for KP10.

**Figure 3:**
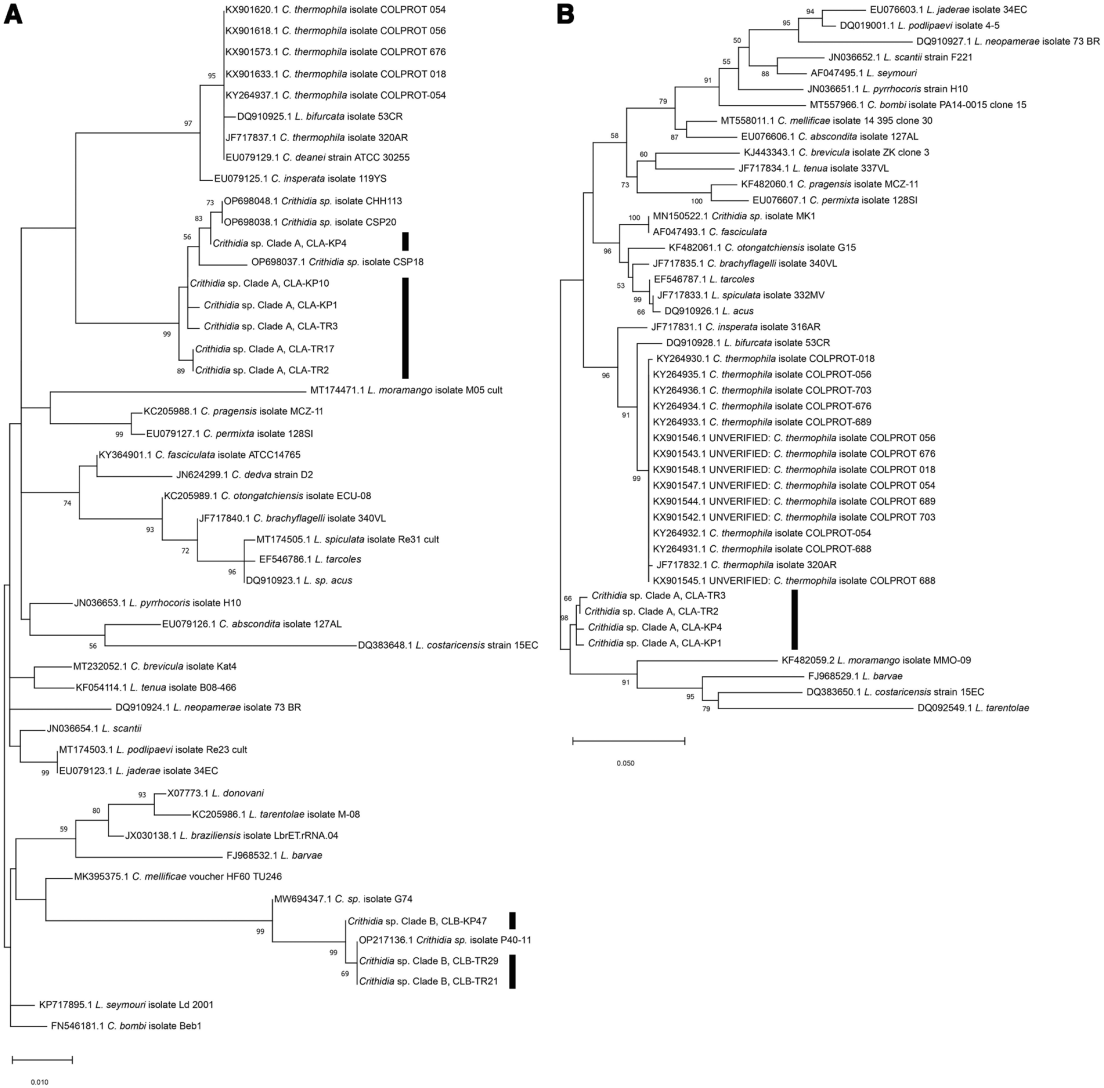
**(A)** Maximum Likelihood tree based on SSU rRNA sequences of *Crithidia* spp., *Leishmania* spp. and *Leptomonas* spp. **(B)** Maximum Likelihood tree based on gGAPDH sequences of *Crithidia* spp., *Leishmania* spp., and *Leptomonas* spp. Bootstrap values are shown at each node. Bootstrap values of ≥70% correspond to supported clades. Nodes with Bootstrap values of <50% are not shown.

Phylogenetic analysis of the SSU rRNA sequences demonstrated that representative sequences from the group of 19, namely from insects TR2, TR3, KP1, TR17, KP4, and KP10 clustered together into one distinct clade with 99% bootstrap values (K2P 0.19–0.77%), designated here as *Crithidia* sp. Clade A (Figure 3A). The corresponding isolates were thus given strain designations *Crithidia* sp. CLA-TR2, *Crithidia* sp. CLA-TR3, and so on. Four of these, CLA-TR2, CLA-TR3, CLA-KP1 and CLA-KP4, were the four isolates successfully isolated into culture. Representatives of the group of 5 from insects TR21, TR29, and KP47 also clustered together with 99% bootstrap values (K2P 0–1.35%), and were designated as *Crithidia* sp. Clade B. The availability of cultures for CLA-TR2, CLA-TR3, CLA-KP1, and CLA-KP4 enabled additional DNA extractions and analysis of their gGAPDH sequences. These gGAPDH sequences also clustered together with 98% bootstrap values (K2P 0.31–0.77%) (Figure 3B). Amongst available sequences they were most similar to those of *Cr. fasciculata* (GenBank accession number AF047493.1) and *Cr. brachyflagelli* (GenBank accession number JF717835.1) with 94.39–94.61% identity (Table 1). However, they were almost as close to a clade of *Cr. thermophila* sequences (K2P 3.77–4.25%), like what was observed in the SSU rRNA analysis (Figure 3A).

In summary, from Tha Ruea seven female *C. peregrinus* were found infected with *Crithidia* sp. Clade A organisms and four females with *Crithidia* sp. Clade B organisms. Successful cultures were obtained only from insects TR2 and TR3, both Clade A organisms (Table 1). In this area the infection rate of *L. martiniquensis* in the 50 parous *C. peregrinus* was 2%, represented by one insect (TR17) also co-infected with *Crithidia* sp. Clade A organisms. In Khuan Phang, twelve females were infected with *Crithidia* sp. Clade A organisms and one female with *Crithidia* sp. Clade B organisms. In this area the infection rate of the *C. peregrinus* with *L. martiniquensis* was 6%, with one insect (TR10) again co-infected with *Crithidia* sp. Clade A organisms, but also in two other insects (KP17 and KP19) that only contained *Leishmania*. We also successfully cultured two *Crithidia* sp. Clade A from two *C. peregrinus* (KP1 and KP4) (Table 1).

### Morphological and ultrastructural analyses of *Crithidia* sp. CLA-KP1 strain

**Figure 4:**
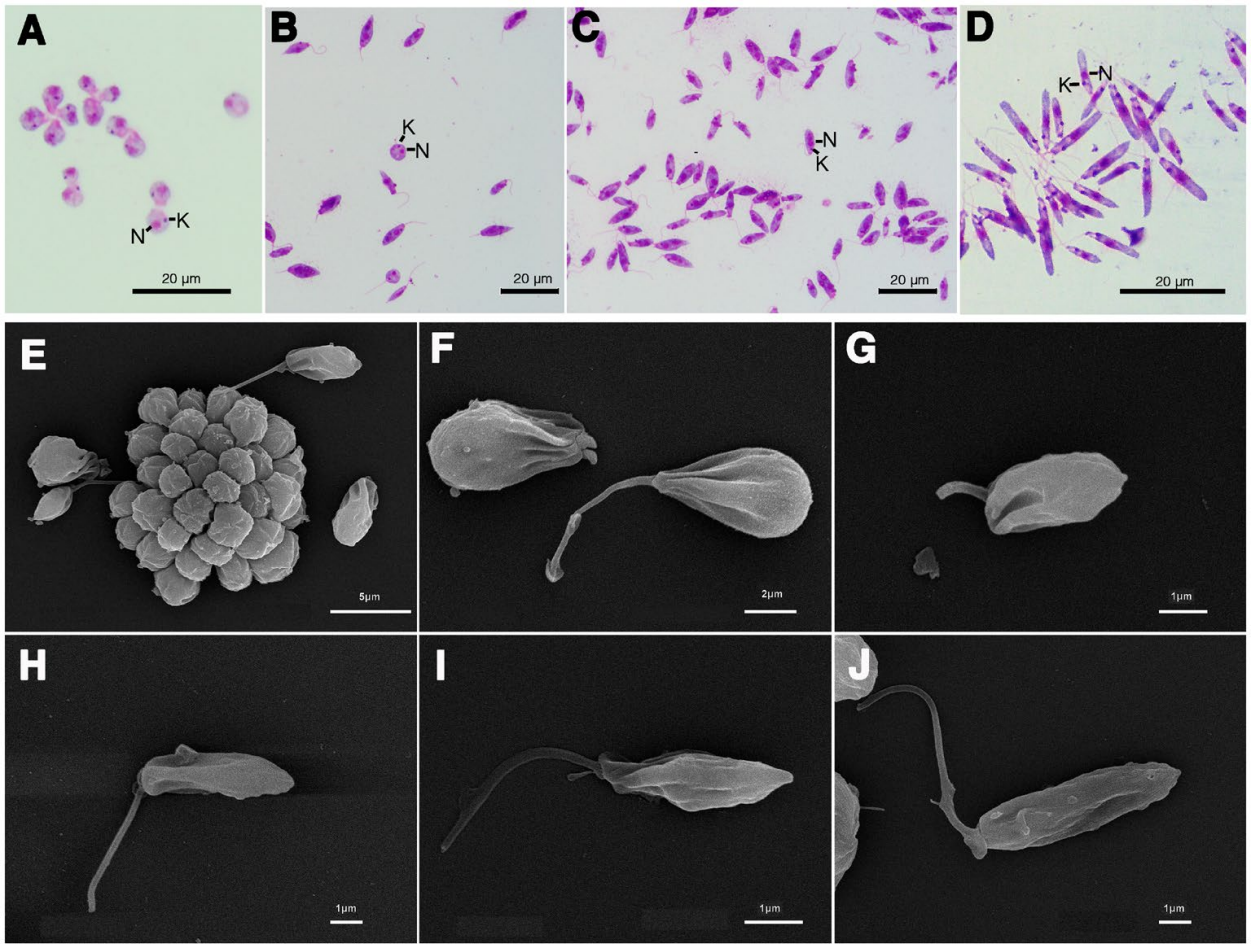
Light microscopy **(A–D)** and SEM **(E–J)** of *Crithidia* sp. CLA-KP1 strain cultured in SIM complete at 26°C. **(A)** Giemsa-stained haptomonad forms. **(B,C)** Giemsa-stained choanomastigotes and promastigotes. **(D)** Giemsa-stained nectomonad forms. **(E)** A rosette form, adherent, non-motile form. **(F)** Haptomonad form and choanomastigote with flagellum. **(G–I)** Promastigotes with different length of flagellum. **(J)** Nectomonad form. *n*, nucleus; k, kinetoplast.

**Figure 5:**
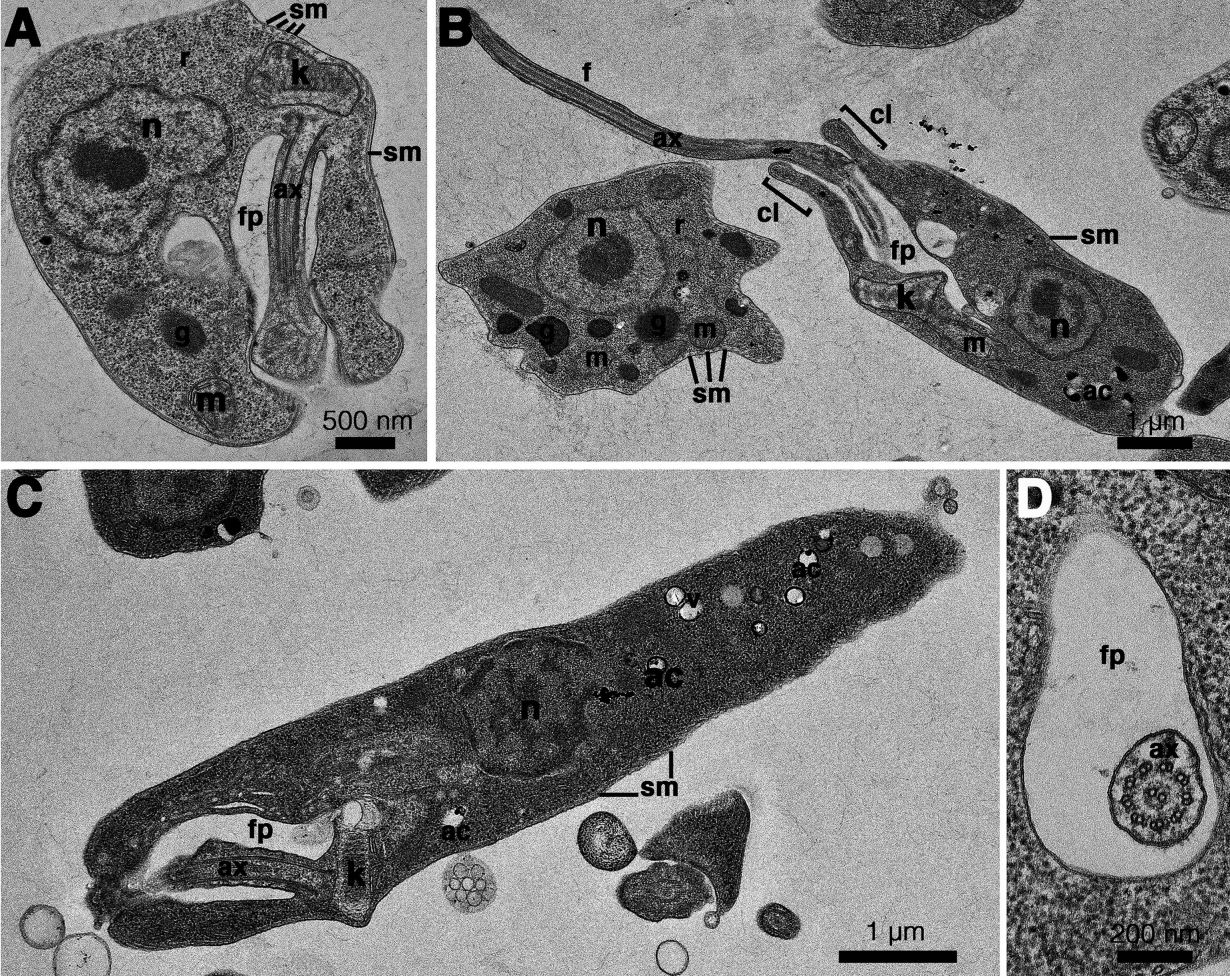
Transmission electron microscopy images of *Crithidia* sp. CLA-KP1 strain cultured in SIM complete at 26°C. **(A)** Longitudinal section of a haptomonad form **(B,C)** Longitudinal section of promastigotes. **(D)** Cross section of a promastigote showing a flagellum with 9 × 2 + 2 axonemal pattern in the flagellar pocket. ac, acidocalcisome; ax, axoneme; f, flagellum; fp, flagellar pocket; g, glycosome; k, kinetoplast; m, mitochondrion; n, nucleus; r, ribosome; sm, subpellicular microtubules; v, vesicles; cl, collar-like extension.

The results of the phylogenetic analysis revealed *Crithidia* sp. Clade A organisms to be related to *Cr. thermophila*, an unusual thermotolerant trypanosomatid. To examine whether Clade A organisms might have similar properties further experiments were performed on strain CLA-KP1. Morphological and ultrastructural analyses of cultures in SIM complete at 26°C revealed two distinct morphotypes, choanomastigotes with rounded posterior ends and promastigotes with elongated posterior ends (Figures 4, 5). A wide collar-shaped reservoir (collar-like extension) surrounding the anterior end through which a single flagellum emerges was noted in both morphotypes (Figures 4A,B, 5B). This single flagellum arose from a basal body (kinetosome) located next to a kinetoplast (Figures 5A–C) and was surrounded by the flagellar pocket (Figure 5). The flagellum exhibited nine pairs of peripheral microtubules surrounding two central microtubules, the typical 9 × 2 + 2 axonemal pattern (Figure 5D). The kinetoplast was located just beyond the basal body, anterior or parallel to the nucleus. The nucleus was oval located in the middle of the cell body with visible accumulations of chromatin. Some other structures, such as acidocalcisomes, glycosomes and ribosomes, were observed (Figures 5A–C). Different forms of the choanomastigotes were observed, including those without a free flagellum (haptomonad forms) (Figures 4A–F, 5A) and others with a free flagellum protruding from the flagellar pocket at the anterior end of the cells (Figures 4B–F). A form of choanomastigote comprising attached clusters of cells (Rosette form) could be observed at the bottom of culture flasks (Figures 4A–E). Promastigotes were slender in shape with a free flagellum at the anterior end of the cells. In addition, morphological differences in some details, such as body width, body length, and flagellum length were observed (Figures 4G–J). Comparative morphological measurements of the choanomastigotes and promastigotes determined under LM are shown in Table 2. Promastigotes with the longest body and flagellum length known as a nectomonad form were noted (Figures 4D–J and Table 2). A single layer of subpellicular microtubules was observed around the body except for the membrane inside the area of the flagellar pocket (Figures 5A–C).

**Table 2:** Morphometry of *Crithidia* sp. CLA-KP1 strain cultured in SIM complete at 26°C.

Morphological feature	Choanomastigotes		Promastigotes	
	Haptomonad form (50)	With flagellum (50)	With flagellum (50)	Nectomonad form (50)
Cell body length (μm)	4.76 ± 0.75	5.5 ± 0.95	7.85 ± 1.6	10.69 ± 1.11
Cell body width (μm)	3.79 ± 0.56	3.9 ± 0.73	3.17 ± 0.72	1.65 ± 0.32
Anterior end to kinetoplast distance (μm)	3.27 ± 0.69	3.48 ± 0.94	7.00 ± 1.27	7.56 ± 1.17
Anterior end to nucleus distance (μm)	2.74 ± 0.67	2.77 ± 0.66	5.08 ± 1.02	9.01 ± 1.16
Flagellum length (μm)	–	3.12 ± 1.77	5.49 ± 0.88	9.5 ± 3.22

### *In vitro* growth of *Crithidia* sp. CLA-KP1 strain at 26 and 37°C

**Figure 6:**
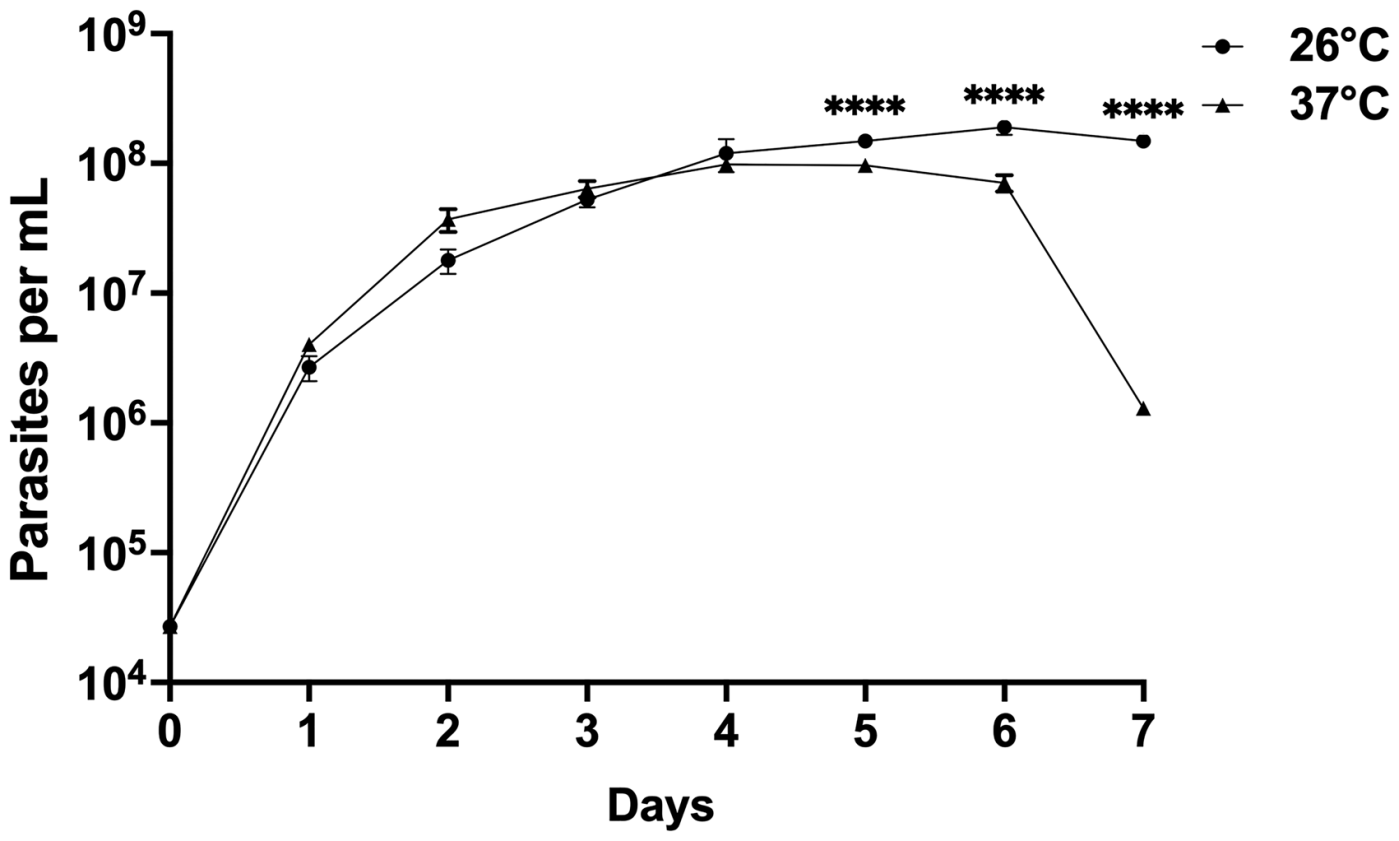
Growth curves of *Crithidia* sp. CLA-KP1 strain cultured in SIM complete for 7 days at 26 and 37°C. Statistically significant differences between 26 and 37°C are indicated as ****p < 0.0001.

CLA-KP1 organisms were cultured at 26 and 37°C with an initial cell density of 4 × 10^4^ cells/ml and observed daily for their growth for seven successive days. On the first 4 days, at both temperatures, similar growth patterns were observed. The parasites entered the exponential growth phase after 1 day of the culture and showed rapid multiplication (day 1 to day 4) with a doubling time of approximately 13.5 and 15.6 h for 26°C and 37°C, respectively. After 4 days of culture, at 26°C, the cultures entered the stationary phase and reached their peak on day 6 with a density of 1.9 × 10^8^ cells/ml. The cultures remained in this phase to the last day of the experiments. At 37°C, CLA-KP1 reached maximum growth on day four with a density of 9.8 × 10^7^ cells/mL, remained in stationary phase for 2 days, then the population dropped dramatically after 6 days of culture until the end of the experiment with a density of 1.0 × 10^6^ cells/ml. The average parasite density at 26°C was significantly greater than 37°C after 5 days of culture (Figure 6 and Supplementary Table S2). Both morphotypes were found in the cultures at both temperatures. However, the predominant morphotype at 26°C was promastigotes, whereas at 37°C choanomastigotes were mainly observed.

### *In vitro* infection and multiplication of *Crithidia* sp. CLA-KP1 strain in mouse macrophages

**Figure 7:**
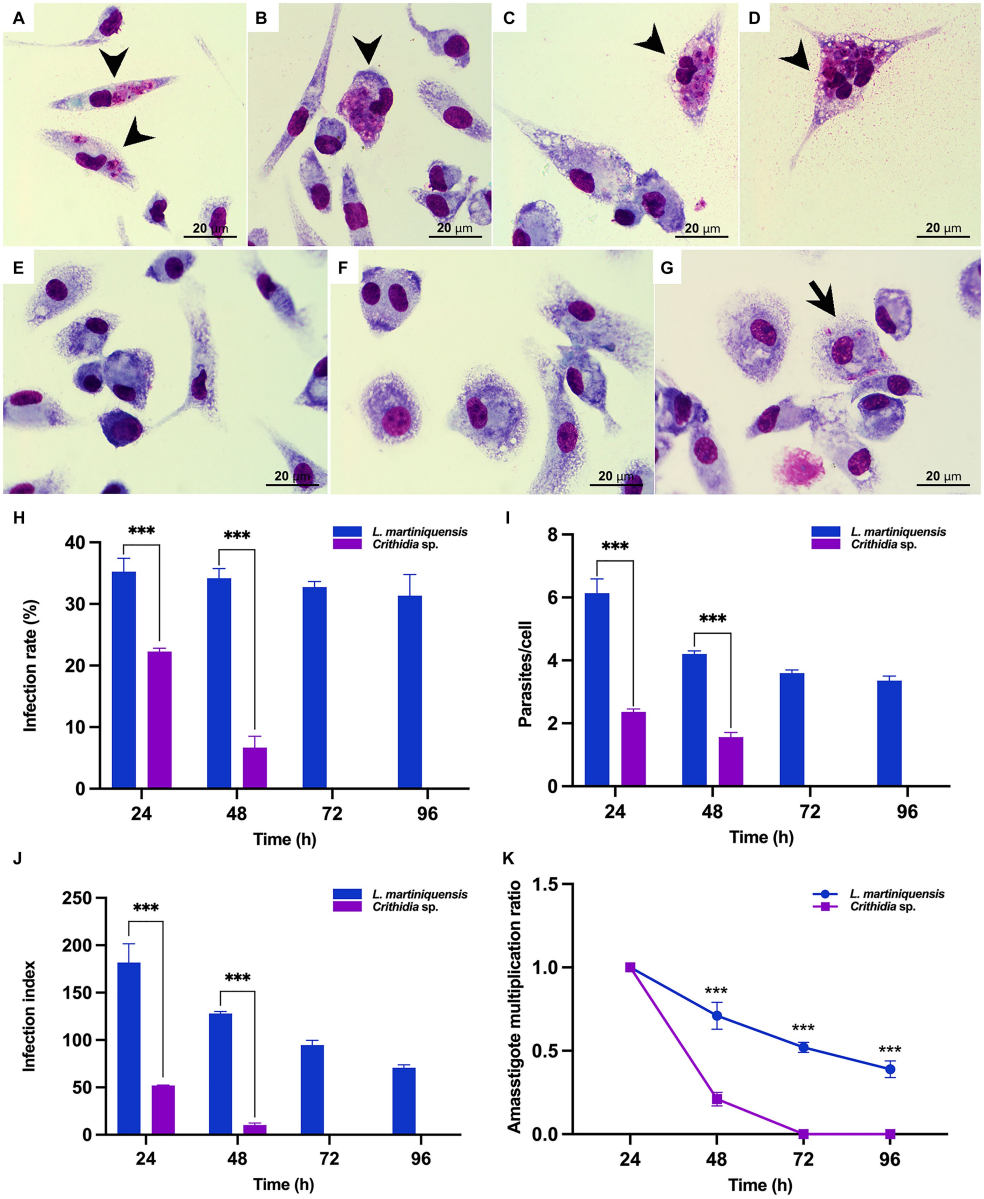
*In vitro* infection of *Crithidia* sp. CLA-KP1 in PEMs. **(A,B)** 24 h after infection. **(C,D)** 48 h after infection. **(E)** 72 h after infection. **(F)** 96 h after infection. **(G)** *L. martiniquensis* infection at 96 h (control). **(H)** Infection rate. **(I)** Average number of intracellular parasites per macrophage. **(J)** Infection index. **(K)** Amastigote multiplication ratio. Results are expressed as the means ± standard deviation from three different experiments run in duplicate. Statistically significant differences between CLA-KP1 and *L. martiniquensis* are indicated as ***p < 0.001.

The ability of CLA-KP1 to infect and multiply in PEMs of BALB/c mice was determined and compared with *L. martiniquensis* (Figure 7). *Crithidia* sp. CLA-KP1 could infect the PEMs and was found at 24 and 48 h but no parasites were observed at 72 and 96 h. At both time points, the infection rate, the average number of intracellular parasites per cell, and the infection index of CLA-KP1 were statistically significantly lower than that of *L. martiniquensis* (Figures 7H–J). At 24 h post-infection, the percentage of PEMs infected with CLA-KP1 was 22.27 ± 0.55 and dramatically decreased to 6.68 ± 1.85 at 48 h, which differed from the stable infection rate in *Leishmania* parasites at the same time points. The average number of intracellular CLA-KP1 parasites within infected PEMs was 2.35 ± 0.09 and 1.56 ± 0.17 parasites/cell at 24 and 48 h, respectively. At both time points, the average number of intracellular parasites/macrophage was approximately 2.6 times lower than that of *L. martiniquensis*. The infection index of CLA-KP1 was 51.97 ± 0.45 and 10.25 ± 2.10 at 24 h and 48 h, respectively, which was lower than that of the *Leishmania* parasites approximately 3.5 and 12.5 times, respectively. The intracellular parasite multiplication ratio of CLA-KP1 was only calculated at 48 h post-infection (0.21) because after that the parasites disappeared. A decrease in the intracellular parasite multiplication ratio was also observed in *L. martiniquensis* after 24 h post-infection, however, the *Leishmania* parasites were still be found in the PEMs at the end of the experiment (96 h) (Figure 7 and Supplementary Table S3). In summary, these results demonstrated that *Crithidia* sp. CLA-KP1 could infect and persist in the PEMs up to 48 h but could not multiply within them.

## Discussion

Evidence that biting midges can act as competent vectors of *L. martiniquensis* and *L. orientalis* has been experimentally demonstrated *in vivo* (Chanmol et al., 2019; Becvar et al., 2021), however, the natural vectors that are responsible for the transmission of leishmaniasis caused by both *Leishmania* species remain unknown. Here, we show the potential for *C. peregrinus* to act as a vector for *L. martiniquensis* in Thailand. Although the number of the biting midges collected was low, *C. peregrinus* was the prominent midge species found in Tha Ruea and Khuan Phang. Due to the collection of the midges performed only in one month in the dry season, the species composition of *Culicoides* species found in both areas was less than the previous reports in other provinces in Thailand (Jomkumsing et al., 2021; Sunantaraporn et al., 2021, 2022; Songumpai et al., 2022). The life cycle of *Culicoides* biting midges varies from 14 days to longer than one year since the development of each *Culicoides* species depends on latitude, climate temperature, and surrounding environmental conditions (Mellor et al., 2000; Mullen, 2009), therefore, different species of *Culicoides* are found in different locations and seasons. However, three species, *C. peregrinus* and *C. oxystoma* and *C. mahasarakhamense*, were found in both locations studied corresponding to a previous report that DNA of *L. martiniquensis* has been detected in these three *Culicoides* species and some other species, *C. huffi*, *C. fordae*, and *C. fulvus*, collected from Songkhla Province, southern Thailand (Songumpai et al., 2022).

One of the key criteria used to incriminate vectors of leishmaniasis is that the vector species supports the development of parasite life stages after the infecting blood meal has been digested through to the transmissible infective metacyclic stage (Killick-Kendrick, 1999; Lawyer and Perkins, 2000). Thus, the presence of metacyclic forms usually accompanied by other promastigote forms in the anterior midgut or stomodeal valve of the collected insects is important evidence to confirm that the insect is a natural vector of the *Leishmania* parasites (Killick-Kendrick, 1999; Ready, 2013). In the current study, various promastigote forms, namely procyclic promastigotes, nectomonad promastigotes, and leptomonad promastigotes, of the *Leishmania* parasites were observed in the foregut of four specimens of *C. peregrinus*. However, no metacyclic promastigotes were found in the Giemsa-stained slides. However, the presence of parasites in the foregut in the absence of bloodmeal remnants indicate these are established infections. The video record of the midge, insect code KP10, showed the movement of the parasites in the foregut, which was similar to the movement of *Leishmania* parasites in the foregut of sandflies (Falcão de Oliveira et al., 2017). The results of molecular identification using two molecular targets, ITS1 and 3’UTR *HSP70* type I, confirmed that the *Leishmania* parasites found in the *C. peregrinus* were *L. martiniquensis*. Altogether, these findings support *C. peregrinus* as a natural vector of *L. martiniquensis*, but as yet do not prove this definitively. The occurrence of *L. martiniquensis-Crithidia* co-infections in two out of the four *Leishmania*-positive midges was interesting, but its significance, if any, is not clear. This may simply be co-incidental, but the possibility that the organisms may interact in some way is worth exploring in future studies.

The infection rates of *L. martiniquensis* in these study areas (2 and 6%) is typical of endemic areas, where only a small proportion of collected vectors are usually found harboring parasites. A recent study focused on the residences of two recently diagnosed visceral leishmaniasis patients in Songkhla Province, Thailand, indicated a local infection rate of 21.2% in *Culicoides* midges, including *C. peregrinus* (Songumpai et al., 2022), but no live dissections were performed. Even though no active case was present in our study areas, the infection rate of the parasites in the insects indicated the presence of the parasites circulating in the wild. It implies that vector(s) and animal reservoir host(s) of *L. martiniquensis* may be present in these locations serving the parasite’s life cycle. No *L. orientalis* DNA or parasites were found in the insects collected in this study, but further investigation in other seasons and areas that have active cases should be carried out.

To investigate whether the biting midges were anthropophilic or not, engorged females were subjected to blood meal analysis. Cow blood DNA was only detected in all collected engorged *C. peregrinus* females confirming the predominantly zoophilic behavior of *C. peregrinus* previously reported (Sunantaraporn et al., 2021, 2022; Kar et al., 2022; Songumpai et al., 2022). In the engorged female samples, no DNAs of *Leishmania* or other trypanosomatids were found. As the number of samples in our study was low, more samples of engorged females of *Culicoides* species are required for further investigation of the anthropophilic and/or zoophilic behaviors of the biting midges. Blood meal identification of the midges in the endemic areas would also help investigate reservoir hosts of autochthonous leishmaniasis in Thailand. For definitive vector incrimination transmission by bite needs to be demonstrated, which could be attempted in the field with natural hosts or with wild-caught naturally infected midges in the laboratory. However, neither of these are technically feasible at present. Our preferred approach is to establish a lab colony of *C. peregrinus* for transmission experiments of *L. martiniquensis* to mice or hamsters. The experimental results proving that *C. peregrinus* could transmit the parasites to mice or hamsters would be implied for the transmission of *L. martiniquensis* from naturally infected *C. peregrinus* to the reservoir hosts.

In this study, no *Leishmania* parasites were successfully isolated into cultures. However, this is a challenging procedure, because although dissection and examination for trypanosomatids under a light microscope was performed using sterile techniques, contamination still occurred from bacteria and fungi present inside the digestive tracts of the midges. Also, the amount of each positive sample was limited due to dividing the sample into three parts for Giemsa’s staining, cultivation, and gDNA extraction. These problems made it difficult to culture *Leishmania* parasites from the dissected insects. A biphasic Novy, McNeil, Nicolle (NNN) culture medium (Nicolle, 1908) containing penicillin–streptomycin and gentamicin was used to establish cultures of *L. macropodum* from midges of the genus *Forcipomyia* (Diptera: Ceratopogonidae) (Dougall et al., 2011). Adding antibiotics such as penicillin–streptomycin and gentamicin may help getting rid of some bacteria but not for fungi. Barratt and colleagues (Barratt et al., 2017) have been successfully isolated a novel trypanosomatid, *Zelonia australiensis* from a black fly, *Simulium dycei*, by serial dilution to get rid of fungi but only if the parasite cells outnumbered the fungi in the cultures (Barratt et al., 2017). Using other media such as the biphasic NNN with antibiotics and performing serial dilution may allow to obtain pure promastigote cultures easily. Recently, our study regarding Amphotericin B (AmpB)-resistant *L. martiniquensis* parasites has suggested that the resistant strains could be more efficiently transmitted and maintained in asymptomatic hosts longer than susceptible ones (Mano et al., 2023). Thus, the isolation of parasites from insects is required to investigate not only the vector competence of the insects but also the prevalence of the AmpB-resistant phenotype parasites in natural insect populations. Furthermore, the insect-isolated parasites would be useful sources for genetic analyses to intensively investigate the mechanisms underlying genetic exchange of the parasites in the insects as the sexual cycle of *Leishmania* parasites has been largely confined to promastigotes developing in the midgut of the vectors (Sadlova et al., 2011; Inbar et al., 2013; Ferreira and Sacks, 2022).

Unlike *Leishmania* parasites, monoxenous trypanosomatids, insect-restricted parasites, are more easily isolated and cultured in the laboratory (Lukeš and Votýpka, 2020). Here, two clades of novel *Crithidia* spp. were identified and four isolates of *Crithidia* sp. Clade A were obtained. Notably, choanomastigotes and promastigotes of both novel *Crithidia* spp. Clades were found in the hindgut of the dissected *C. peregrinus* corresponding to the reported developmental habitat of *Crithidia* spp. in insects (Frolov et al., 2021). The morphology of the forms of *Crithidia* sp. CLA was similar to those described in *Cr. mellificae* (Schwarz et al., 2015) and *Cr. fasciculata* (Filosa et al., 2019). TEM analysis showed typical morphological features of trypanosomatids: an oval nucleus; a kinetoplast; a flagellum with 9 × 2 + 2 axonemal pattern in the flagellar pocket; glycosomes; a reticulated mitochondrion with numerous cristae; and subpellicular microtubules. *Cr. thermophila* is reclassified and synonymous with *Cr. luciliae thermophila* and *Cr. confusa* (Ishemgulova et al., 2017). Only two forms, choanomastigotes and promastigotes with flagellum of *Cr. thermophila* grown in liquid Brain Heart Infusion (BHI) medium at 23°C have been reported (Ishemgulova et al., 2017). Comparing to *Cr. thermophila,* the size of choanomastigotes and promastigotes with flagellum of CLA-KP1 was slightly larger than that of *Cr. thermophila* (Ishemgulova et al., 2017) supporting that they are different species.

The ability to survive at human body temperature and multiply in mammalian macrophage cells is a hallmark of dixenous parasites such as *Leishmania* but these features are not common in monoxenous trypanosomatids. However, certain monoxenous trypanosomatids have been reported in warm-blooded animals including humans. *Cr. mellificae* can infect not only insects but also various mammals (Dario et al., 2021). In addition, strains of *Crithidia* sp. related to *Cr*. *fasciculata* have been isolated from lesions in an immunocompetent patient with no underlying diseases (Maruyama et al., 2019) or with leishmaniasis (Ghobakhloo et al., 2019; Kostygov et al., 2019; Rogerio et al., 2023). Moreover, thermotolerance to high temperatures has been demonstrated *in vitro* in *Cr. thermophila* (34°C) (Ishemgulova et al., 2017), *Cr. mellificae* (36–37°C) (Dario et al., 2021), and a *Crithidia* sp. related to *Cr*. *fasciculata* (37°C) (Ghobakhloo et al., 2019). In our study, *Crithidia* sp. CLA-KP1 was able to grow and survive at 37°C in culture for at least 7 days and the density of the cells at day 7 was still high (1.0 × 10^6^ cells/mL), although the average parasite density at 26°C was significantly greater than 37°C after 5 days of culture. In addition, *Crithidia* sp. CLA-KP1 could infect and persist appear in the PEMs for up to 48 h. The results suggested that *Crithidia* sp. CLA-KP1 is a thermotolerant monoxenous trypanosomatid that could live in a wide range of temperature, 26–37°C.

Ghobakhloo and colleagues (Ghobakhloo et al., 2019) have demonstrated that clinical isolates of *Crithidia* sp. related to *Cr*. *fasciculata* are able to infect THP-1 and J774 cells and transform back to promastigotes in culture media but the percentage of infection is much less than that of the clinical isolate of *L. major* (Ghobakhloo et al., 2019). Another opportunistic trypanosomatid that co-infects in leishmaniasis patients is *Le. seymouri*. The *Le. seymouri* parasites isolated from clinical samples can grow well at 35°C but are unable to infect mammalian macrophages *in vitro* either alone or in co-infection with *Leishmania* parasites (Kraeva et al., 2015). Although *Crithidia* sp. CLA-KP1 was isolated from insects, further investigation of *in vitro* infection using other cell lines such as THP-1, J774, and BMMɸ cells should be performed. Comparative analyses of genomic and transcriptomic profiles of these thermotolerant monoxenous trypanosomatids and dixenous parasites would throw light on the adaptations of trypanosomatids to elevated temperature and tracking the evolution of parasitism of dixenous trypanosomatids.

In conclusion, here we provide microscopic analyses together with molecular identification of naturally occurring infections of *L. martiniquensis* in *C. peregrinus* for the first time. The findings strongly support this biting midge species as a potential vector of *L. martiniquensis*. However, further isolation and characterization of parasites from more biting midges, including other species, are required to search for more natural infections of both *L. martiniquensis* and *L. orientalis*. Also, further investigation of the anthropophilic and/or zoophilic behaviors of the biting midge, *C. peregrinus*, is needed to investigate reservoir hosts of autochthonous leishmaniasis in Thailand. Finally, for definitive vector incrimination transmission by bite needs to be demonstrated. Four strains of *Crithidia* sp. CLA were isolated from *C. peregrinus* and characterized for the first time. The CLA-KP1 strain could infect PEMs but they could not multiply suggesting that it was a thermotolerant monoxenous trypanosomatid capable of surviving in a wide range of temperatures (26–37°C). Analysis of genome sequences of all strains of *Crithidia* sp. CLA will be performed to compare the evolutionary relationship between this newly isolated trypanosomatid and other related parasites. A description of the detailed molecular characterization and taxonomic assignment will be provided in the future.

## Data availability statement

The datasets presented in this study can be found in online repositories. The names of the repository/repositories and accession number(s) can be found in the article/Supplementary material.

## Ethics statement

The use of animals in this study was approved by the animal research ethics committee of Chulalongkorn University Animal Care and Use Protocol (CU-ACUP), Faculty of Medicine, Chulalongkorn University, Bangkok, Thailand (COA No. 023/2564 and COA No. 011/2564). The study was conducted in accordance with the local legislation and institutional requirements.

## Author contributions

NJ and PS: conceptualization, methodology, and formal analysis. SK, NJ, CM, TP, RA, TY, UP, and AJ: investigation. SK, PS, and NJ: visualization. NJ, SK, TP, and CM: writing – original draft preparation. NJ and PB: writing-review and editing. NJ: funding acquisition. All authors have read and agreed to the published version of the manuscript.

## Funding

This work was supported by the 90th Anniversary of Chulalongkorn University Fund (Ratchadaphiseksomphot Endowment Fund) (grant number GCUGR1125651016M).

## Conflict of interest

The authors declare that the research was conducted in the absence of any commercial or financial relationships that could be construed as a potential conflict of interest.

## Publisher’s note

All claims expressed in this article are solely those of the authors and do not necessarily represent those of their affiliated organizations, or those of the publisher, the editors and the reviewers. Any product that may be evaluated in this article, or claim that may be made by its manufacturer, is not guaranteed or endorsed by the publisher.

## Footnotes

^1^ https://blast.ncbi.nlm.nih.gov/Blast.cgi

## References

[ref1] Anugulruengkitt S. Songtaweesin W. N. Thepnarong N. Tangthanapalakul A. Sitthisan M. Chatproedprai S.. (2022). Case report: simple nodular cutaneous leishmaniasis caused by autochthonous *Leishmania* (*Mundinia*) *orientalis* in an 18-month-old girl: the first pediatric case in Thailand and literature review. Am. J. Trop. Med. Hyg. 108, 44–50. doi: 10.4269/ajtmh.22-0385, PMID: 36410322 PMC9833080

[ref2] Barratt J. Kaufer A. Peters B. Craig D. Lawrence A. Roberts T.. (2017). Isolation of novel Trypanosomatid, *Zelonia australiensis* sp. nov. (Kinetoplastida: Trypanosomatidae) provides support for a gondwanan origin of dixenous parasitism in the *Leishmaniinae*. PLoS Negl. Trop. Dis. 11:e0005215. doi: 10.1371/journal.pntd.0005215, PMID: 28081121 PMC5230760

[ref3] Becvar T. Vojtkova B. Siriyasatien P. Votypka J. Modry D. Jahn P.. (2021). Experimental transmission of *Leishmania* (*Mundinia*) parasites by biting midges (Diptera: Ceratopogonidae). PLoS Pathog. 17:e1009654. doi: 10.1371/journal.ppat.1009654, PMID: 34115806 PMC8221790

[ref4] Bernotienė R. Iezhova T. A. Bukauskaitė D. Chagas C. R. F. Kazak M. Valkiūnas G. (2020). Development of *Trypanosoma everetti* in *Culicoides* biting midges. Acta Trop. 210:105555. doi: 10.1016/j.actatropica.2020.105555 32473117

[ref5] Cecílio P. Cordeiro-da-Silva A. Oliveira F. (2022). Sand flies: basic information on the vectors of leishmaniasis and their interactions with *Leishmania* parasites. Commun. Biol. 5:305. doi: 10.1038/s42003-022-03240-z, PMID: 35379881 PMC8979968

[ref6] Chanmol W. Jariyapan N. Somboon P. Bates M. D. Bates P. A. (2019). Development of *Leishmania orientalis* in the sand fly *Lutzomyia longipalpis* (Diptera: Psychodidae) and the biting midge *Culicoides soronensis* (Diptera: Ceratopogonidae). Acta Trop. 199:105157. doi: 10.1016/j.actatropica.2019.105157 31491400

[ref7] Chusri S. Thammapalo S. Chusri S. Thammapalo S. Silpapojakul K. Siriyasatien P. (2014). Animal reservoirs and potential vectors of *Leishmania siamensis* in southern Thailand. Southeast Asian J. Trop. Med. Public Health. 45, 13–19. 24964648

[ref8] Dario M. A. Lisboa C. V. Silva M. V. Herrera H. M. Rocha F. L. Furtado M. C.. (2021). *Crithidia mellificae* infection in different mammalian species in Brazil. Int. J. Parasitol. Parasites Wildl. 15, 58–69. doi: 10.1016/j.ijppaw.2021.04.003, PMID: 33981571 PMC8085711

[ref9] Dougall A. M. Alexander B. Holt D. C. Harris T. Sultan A. H. Bates P. A.. (2011). Evidence incriminating midges (Diptera: Ceratopogonidae) as potential vectors of *Leishmania* in Australia. Int. J. Parasitol. 41, 571–579. doi: 10.1016/j.ijpara.2010.12.008, PMID: 21251914

[ref10] Dyce A. L. (1996). *Culicoides paragarciai*, a new Ornatus group species from Papua New Guinea and the Solomon Islands (Diptera: Ceratopogonidae). Aust. J. Entomol. 35, 313–318. doi: 10.1111/j.1440-6055.1996.tb01410.x

[ref11] Dyce A.L. Bellis G.A. Muller M.J. (2007). Pictorial atlas of Australasian *Culicoides* wings (Diptera: Ceratopogonidae). Canberra, Australia: Australian Biological Resources Study

[ref12] Falcão de Oliveira E. Oshiro E. T. Fernandes W. S. Murat P. G. de Medeiros M. J. Souza A. I.. (2017). Experimental infection and transmission of *Leishmania* by *Lutzomyia cruzi* (Diptera: Psychodidae): aspects of the ecology of parasite-vector interactions. PLoS Negl. Trop. Dis. 11:e0005401. doi: 10.1371/journal.pntd.0005401, PMID: 28234913 PMC5342273

[ref13] Ferreira T. R. Sacks D. L. (2022). Experimental hybridization in *Leishmania*: tools for the study of genetic exchange. Pathogens 11:580. doi: 10.3390/pathogens11050580, PMID: 35631101 PMC9144296

[ref14] Filosa J. N. Berry C. T. Ruthel G. Beverley S. M. Warren W. C. Tomlinson C.. (2019). Dramatic changes in gene expression in different forms of *Crithidia fasciculata* reveal potential mechanisms for insect-specific adhesion in kinetoplastid parasites. PLoS Negl. Trop. Dis. 13:e0007570. doi: 10.1371/journal.pntd.0007570, PMID: 31356610 PMC6687205

[ref15] Folmer O. Black M. Hoeh W. Lutz R. Vrijenhoek R. (1994). DNA primers for amplification of mitochondrial cytochrome c oxidase subunit I from diverse metazoan invertebrates. Mol. Mar. Biol. Biotechnol. 3, 294–299. PMID: 7881515

[ref16] Frolov A. O. Kostygov A. Y. Yurchenko V. (2021). Development of monoxenous Trypanosomatids and Phytomonads in insects. Trends Parasitol. 37, 538–551. doi: 10.1016/j.pt.2021.02.004, PMID: 33714646

[ref17] Ghobakhloo N. Motazedian M. H. Naderi S. Ebrahimi S. (2019). Isolation of *Crithidia* spp. from lesions of immunocompetent patients with suspected cutaneous leishmaniasis in Iran. Tropical Med. Int. Health 24, 116–126. doi: 10.1111/tmi.13042, PMID: 29446852

[ref18] Ghosh S. Banerjee P. Sarkar A. Datta S. Chatterjee M. (2012). Coinfection of *Leptomonas seymouri* and *Leishmania donovani* in Indian leishmaniasis. J. Clin. Microbiol. 50, 2774–2778. doi: 10.1128/JCM.00966-12, PMID: 22622439 PMC3421535

[ref19] Harrup L. E. Laban S. Purse B. V. Reddy Y. K. Reddy Y. N. Byregowda S. M.. (2016). DNA barcoding and surveillance sampling strategies for *Culicoides* biting midges (Diptera: Ceratopogonidae) in southern India. Parasit. Vectors 9:461. doi: 10.1186/s13071-016-1722-z, PMID: 27549137 PMC4994320

[ref20] Hillis D. M. Bull J. J. (1993). An empirical test of bootstrapping as a method for assessing confidence in phylogenetic analysis. Syst. Biol. 42, 182–192. doi: 10.1093/sysbio/42.2.182

[ref21] Inbar E. Akopyants N. S. Charmoy M. Romano A. Lawyer P. Elnaiem D. E.. (2013). The mating competence of geographically diverse *Leishmania major* strains in their natural and unnatural sand fly vectors. PLoS Genet. 9:e1003672. doi: 10.1371/journal.pgen.1003672, PMID: 23935521 PMC3723561

[ref22] Ishemgulova A. Butenko A. Kortišová L. Boucinha C. Grybchuk-Ieremenko A. Morelli K. A.. (2017). Molecular mechanisms of thermal resistance of the insect trypanosomatid *Crithidia thermophila*. PLoS One 12:e0174165. doi: 10.1371/journal.pone.0174165, PMID: 28328988 PMC5362078

[ref23] Jariyapan N. Bates M. D. Bates P. A. (2021). Molecular identification of two newly identified human pathogens causing leishmaniasis using PCR-based methods on the 3′ untranslated region of the heat shock protein 70 (type I) gene. PLoS Negl. Trop. Dis. 15:e0009982. doi: 10.1371/journal.pntd.0009982, PMID: 34847144 PMC8631652

[ref24] Jariyapan N. Daroontum T. Jaiwong K. Chanmol W. Intakhan N. Sor-Suwan S.. (2018). *Leishmania* (*Mundinia*) *orientalis* n. sp. (Trypanosomatidae), a parasite from Thailand responsible for localised cutaneous leishmaniasis. Parasit. Vectors 11:351. doi: 10.1186/s13071-018-2908-3, PMID: 29914526 PMC6006788

[ref25] Jomkumsing P. Surapinit A. Saengpara T. Pramual P. (2021). Genetic variation, DNA barcoding and blood meal identification of *Culicoides Latreille* biting midges (Diptera: Ceratopogonidae) in Thailand. Acta Trop. 217:105866. doi: 10.1016/j.actatropica, PMID: 33607064

[ref26] Kar S. Mondal B. Ghosh J. Mazumdar S. M. Mazumdar A. (2022). Host preference of bluetongue virus vectors, *Culicoides* species associated with livestock in West Bengal, India: potential relevance on bluetongue epidemiology. Acta Trop. 235:106648. doi: 10.1016/j.actatropica.2022.106648 35961406

[ref27] Kasičová Z. Schreiberová A. Kimáková A. Kočišová A. (2021). Blood meal analysis: host-feeding patterns of biting midges (Diptera, Ceratopogonidae, *Culicoides Latreille*) in Slovakia. Parasite 28:58. doi: 10.1051/parasite/2021058, PMID: 34283022 PMC8336726

[ref28] Killick-Kendrick R. (1999). The biology and control of phlebotomine sand flies. Clin. Dermatol. 17, 279–289. doi: 10.1016/s0738-081x(99)00046-2, PMID: 10384867

[ref29] Kostygov A. Y. Butenko A. Yurchenko V. (2019). On monoxenous trypanosomatids from lesions of immunocompetent patients with suspected cutaneous leishmaniasis in Iran. Tropical Med. Int. Health 24, 127–128. doi: 10.1111/tmi.13168, PMID: 30307678

[ref30] Kraeva N. Butenko A. Hlaváčová J. Kostygov A. Myškova J. Grybchuk D.. (2015). *Leptomonas seymouri*: adaptations to the dixenous life cycle analyzed by genome sequencing, transcriptome profiling and co-infection with *Leishmania donovani*. PLoS Pathog. 11:e1005127. doi: 10.1371/journal.ppat.1005127, PMID: 26317207 PMC4552786

[ref31] Kumar S. Stecher G. Li M. Knyaz C. Tamura K. (2018). MEGA X: molecular evolutionary genetics analysis across computing platforms. Mol. Biol. Evol. 35, 1547–1549. doi: 10.1093/molbev/msy096, PMID: 29722887 PMC5967553

[ref32] Kwakye-Nuako G. Mosore M. T. Boakye D. Bates P. A. (2023). Description, biology, and medical significance of *Lishmania* (*Mundinia*) *chancei* n. sp. (Kinetoplastea: Trypanosomatidae) from Ghana and *Leishmania* (*Mundinia*) *procaviensis* N. Sp. (Kinetoplastea: Trypanosomatidae) from Namibia. J. Parasitol. 109, 43–50. doi: 10.1645/22-53 36848641

[ref33] Lawyer P. G. Perkins P. V. (2000). “Leishmaniasis and trypanosomiasis” in Medical Entomology. eds. Eldridge B. F. Edman J. D. (Dordrecht: Springer)

[ref34] Lukeš J. Votýpka J. (2020). Field isolation and cultivation of Trypanosomatids from insects. Methods Mol. Biol. 2116, 3–21. doi: 10.1007/978-1-0716-0294-2_1, PMID: 32221910

[ref35] Mano C. Kongkaew A. Tippawangkosol P. Somboon P. Roytrakul S. Pescher P.. (2023). Amphotericin B resistance correlates with increased fitness *in vitro* and *in vivo* in *Leishmania* (*Mundinia*) *martiniquensis*. Front. Microbiol. 14:1156061. doi: 10.3389/fmicb.2023.1156061, PMID: 37089544 PMC10116047

[ref36] Maruyama S. R. de Santana A. K. M. Takamiya N. T. Takahashi T. Y. Rogerio L. A. Oliveira C. A. B.. (2019). Non-leishmania parasite in fatal visceral Leishmaniasis-like disease. Brazil. *Emerg. Infect. Dis.* 25, 2088–2092. doi: 10.3201/eid2511.181548, PMID: 31625841 PMC6810192

[ref37] Maslov D. A. Lukes J. Jirku M. Simpson L. (1996). Phylogeny of trypanosomes as inferred from the small and large subunit rRNAs: implications for the evolution of parasitism in the trypanosomatid protozoa. Mol. Biochem. Parasitol. 75, 197–205. doi: 10.1016/0166-6851(95)02526-x, PMID: 8992318

[ref38] Mellor P. S. Boorman J. Baylis M. (2000). *Culicoides* biting midges: their role as arbovirus vectors. Annu. Rev. Entomol. 45, 307–340. doi: 10.1146/annurev.ento.45.1.307 10761580

[ref39] Morsy T. A. Schnur L. F. Feinsod F. M. Michael S. A. Saah A. Salama M. M.. (1988). The discovery and preliminary characterization of a novel trypanosomatid parasite from *Rattus norvegicus* and stray dogs from Alexandria, Egypt. Ann. Trop. Med. Parasitol. 82, 437–444. doi: 10.1080/00034983.1988.11812273 3257073

[ref40] Mullen G. R. (2009). Biting midges (Ceratopogonidae). Medical and veterinary entomology. 2nd Edn. ed. Mullen G. R. Durden L. A. (New York, NY: Academic Press), 169–188.

[ref41] Nicolle C. (1908). Culture du parasite du bouton d'Orient. *C. R. Acad*. *Sci*. 146, 842–843.

[ref42] Noyes H. A. Stevens J. R. Teixeira M. Phelan J. Holz P. (1999). A nested PCR for the ssrRNA gene detects *Trypanosoma binneyi* in the platypus and *Trypanosoma* sp. in wombats and kangaroos in Australia. Int. J. Parasitol. 29, 331–339. doi: 10.1016/s0020-7519(98)00167-2, PMID: 10221634

[ref43] Podlipaev S. A. Sturm N. R. Fiala I. Fernandes O. Westenberger S. J. Dollet M.. (2004). Diversity of insect trypanosomatids assessed from the spliced leader RNA and 5S rRNA genes and intergenic regions. J. Eukaryot. Microbiol. 51, 283–290. doi: 10.1111/j.1550-7408.2004.tb00568.x 15218696

[ref44] Podlipaev S. Votýpka J. Jirků M. Svobodová M. Lukes J. (2004). *Herpetomonas ztiplika* n. sp. (Kinetoplastida: Trypanosomatidae): a parasite of the blood-sucking biting midge *Culicoides kibunensis* Tokunaga, 1937 (Diptera: Ceratopogonidae). J. Parasitol. 90, 342–347. doi: 10.1645/GE-156R., PMID: 15165057

[ref45] Posada D. (2008). jModelTest: phylogenetic model averaging. Mol. Biol. Evol. 25, 1253–1256. doi: 10.1093/molbev/msn083 18397919

[ref46] Pothirat T. Tantiworawit A. Chaiwarith R. Jariyapan N. Wannasan A. Siriyasatien P.. (2014). First isolation of *Leishmania* from northern Thailand: case report, identification as *Leishmania martiniquensis* and phylogenetic position within the *Leishmania enriettii* complex. PLoS Negl. Trop. Dis. 8:e3339. doi: 10.1371/journal.pntd.0003339, PMID: 25474647 PMC4256172

[ref47] Pramual P. Jomkumsing P. Piraonapicha K. Jumpato W. (2021). Integrative taxonomy uncovers a new *Culicoides* (Diptera: Ceratopogonidae) biting midge species from Thailand. Acta Trop. 220:105941. doi: 10.1016/j.actatropica.2021.105941, PMID: 33951420

[ref48] Radrova J. Seblova V. Votypka J. (2013). Feeding behavior and spatial distribution of *Culex* mosquitoes (Diptera: Culicidae) in wetland areas of the Czech Republic. J. Med. Entomol. 50, 1097–1104. doi: 10.1603/me13029, PMID: 24180115

[ref49] Rangel D. A. Lisboa C. V. Novaes R. L. M. Silva B. A. Souza R. F. Jansen A. M.. (2019). Isolation and characterization of trypanosomatids, including *Crithidia mellificae*, in bats from the Atlantic Forest of Rio de Janeiro. Brazil*. PLoS Negl. Trop. Dis.* 13:e0007527. doi: 10.1371/journal.pntd.0007527, PMID: 31291252 PMC6619607

[ref50] Ready P. D. (2013). Biology of phlebotomine sand flies as vectors of disease agents. Annu. Rev. Entomol. 58, 227–250. doi: 10.1146/annurev-ento-120811-153557 23317043

[ref51] Rebêlo J. M. Rodrigues B. L. Bandeira M. D. Moraes J. L. Fonteles R. S. Pereira S. R. (2016). Detection of *Leishmania amazonensis* and *Leishmania braziliensis* in *Culicoides* (Diptera, Ceratopogonidae) in an endemic area of cutaneous leishmaniasis in the Brazilian Amazonia. J. Vector Ecol. 41, 303–308. doi: 10.1111/jvec.12227, PMID: 27860021

[ref52] Requena J. M. Chicharro C. García L. Parrado R. Puerta C. J. Cañavate C. (2012). Sequence analysis of the 3′-untranslated region of *HSP70* (type I) genes in the genus *Leishmania*: its usefulness as a molecular marker for species identification. Parasit. Vectors 5:87. doi: 10.1186/1756-3305-5-87, PMID: 22541251 PMC3425316

[ref53] Rogerio L. A. Takahashi T. Y. Cardoso L. Takamiya N. T. de Melo E. V. de Jesus A. R.. (2023). Co-infection of *Leishmania infantum* and a *Crithidia*-related species in a case of refractory relapsed visceral leishmaniasis with non-ulcerated cutaneous manifestation in Brazil. Int. J. Infect. Dis. 133, 85–88. doi: 10.1016/j.ijid.2023.05.012, PMID: 37182549 PMC10330508

[ref54] Sadlova J. Yeo M. Seblova V. Lewis M. D. Mauricio I. Volf P.. (2011). Visualisation of *Leishmania donovani* fluorescent hybrids during early stage development in the sand fly vector. PLoS One 6:e19851. doi: 10.1371/journal.pone.0019851, PMID: 21637755 PMC3103508

[ref55] Schwarz R. S. Bauchan G. R. Murphy C. A. Ravoet J. de Graaf D. C. Evans J. D. (2015). Characterization of two species of Trypanosomatidae from the honey bee *Apis mellifera*: *Crithidia mellificae* Langridge and McGhee, and *Lotmaria passim* n. gen., n. sp. J. Eukaryot. Microbiol. 62, 567–583. doi: 10.1111/jeu.12209, PMID: 25712037

[ref56] Seblova V. Sadlova J. Carpenter S. Volf P. (2012). Development of *Leishmania* parasites in *Culicoides nubeculosus* (Diptera: Ceratopogonidae) and implications for screening vector competence. J. Med. Entomol. 49, 967–970. doi: 10.1603/me12053, PMID: 23025175

[ref57] Singh N. Chikara S. Sundar S. (2013). SOLiD™ sequencing of genomes of clinical isolates of *Leishmania donovani* from India confirm leptomonas co-infection and raise some key questions. PLoS One 8:e55738. doi: 10.1371/journal.pone.0055738, PMID: 23418454 PMC3572117

[ref58] Siripattanapipong S. Leelayoova S. Ninsaeng U. Mungthin M. (2018). Detection of DNA of *Leishmania siamensis* in *Sergentomyia* (*Neophlebotomus*) *iyengari* (Diptera: Psychodidae) and molecular identification of blood meals of sand flies in an affected area, Southern Thailand. J Med Entomol. 55, 1277–1283. doi: 10.1093/jme/tjy069, PMID: 29688539

[ref59] Slama D. Haouas N. Remadi L. Mezhoud H. Babba H. Chaker E. (2014). First detection of *Leishmania infantum* (Kinetoplastida: Trypanosomatidae) in *Culicoides* spp. (Diptera: Ceratopogonidae). Parasit. Vectors 7:51. doi: 10.1186/1756-3305-7-51, PMID: 24460752 PMC3906888

[ref60] Songumpai N. Promrangsee C. Noopetch P. Siriyasatien P. Preativatanyou K. (2022). First evidence of co-circulation of emerging *Leishmania martiniquensis*, *Leishmania orientalis*, and *Crithidia* sp. in *Culicoides* biting midges (Diptera: Ceratopogonidae), the putative vectors for autochthonous transmission in southern Thailand. Trop. Med. Infect. Dis. 7:379. doi: 10.3390/tropicalmed7110379, PMID: 36422930 PMC9696774

[ref61] Spanakos G. Piperaki E. T. Menounos P. G. Tegos N. Flemetakis A. Vakalis N. C. (2008). Detection and species identification of Old World *Leishmania* in clinical samples using a PCR-based method. Trans. R. Soc. Trop. Med. Hyg. 102, 46–53. doi: 10.1016/j.trstmh.2007.05.019, PMID: 17669452

[ref62] Srisuton P. Phumee A. Sunantaraporn S. Boonserm R. Sor-Suwan S. Brownell N.. (2019). Detection of *Leishmania* and *Trypanosoma* DNA in field-caught sand flies from endemic and non-endemic areas of leishmaniasis in southern Thailand. Insects 10:238. doi: 10.3390/insects10080238, PMID: 31382501 PMC6722825

[ref63] Srivarasat S. Brownell N. Siriyasatien P. Noppakun N. Asawanonda P. Rattanakorn K.. (2022). Case report: autochthonous disseminated cutaneous, mucocutaneous, and visceral leishmaniasis caused by *Leishmania martiniquensis* in a patient with HIV/AIDS from northern Thailand and literature review. Am J Trop Med Hyg. 107, 1196–1202. doi: 10.4269/ajtmh.22-0108, PMID: 36375453 PMC9768252

[ref64] Srivastava P. Prajapati V. K. Vanaerschot M. Van der Auwera G. Dujardin J. C. Sundar S. (2010). Detection of *Leptomonas* sp. parasites in clinical isolates of kala-azar patients from India. Infect. Genet. Evol. 10, 1145–1150. doi: 10.1016/j.meegid.2010.07.009, PMID: 20633704 PMC2933273

[ref65] Sriwongpan P. Nedsuwan S. Manomat J. Charoensakulchai S. Lacharojana K. Sankwan J.. (2021). Prevalence and associated risk factors of *Leishmania* infection among immunocompetent hosts, a community-based study in Chiang Rai, Thailand. PLoS Negl. Trop. Dis. 15:e0009545. doi: 10.1371/journal.pntd.0009545, PMID: 34252099 PMC8297947

[ref66] Sunantaraporn S. Hortiwakul T. Kraivichian K. Siriyasatien P. Brownell N. (2022). Molecular identification of host blood meals and detection of blood parasites in *Culicoides Latreille* (Diptera: Ceratopogonidae) collected from Phatthalung province, Southern Thailand. Insects 13:912. doi: 10.3390/insects13100912, PMID: 36292860 PMC9604321

[ref67] Sunantaraporn S. Thepparat A. Phumee A. Sor-Suwan S. Boonserm R. Bellis G.. (2021). *Culicoides Latreille* (Diptera: Ceratopogonidae) as potential vectors for *Leishmania martiniquensis* and *Trypanosoma* sp. in northern Thailand. PLoS Negl. Trop. Dis. 15:e0010014. doi: 10.1371/journal.pntd.0010014, PMID: 34910720 PMC8673663

[ref68] Svobodová M. Dolnik O. V. Čepička I. Rádrová J. (2017). Biting midges (Ceratopogonidae) as vectors of avian trypanosomes. Parasit. Vectors 10:224. doi: 10.1186/s13071-017-2158-9, PMID: 28482865 PMC5423023

[ref69] Svobodová M. Zídková L. Čepička I. Oborník M. Lukeš J. Votýpka J. (2007). *Sergeia podlipaevi* gen. nov., sp. nov. (Trypanosomatidae, Kinetoplastida), a parasite of biting midges (Ceratopogonidae, Diptera). Int. J. Syst. Evol. Microbiol. 57, 423–432. doi: 10.1099/ijs.0.64557-0 17267991

[ref70] Thakur L. Kushwaha H. R. Negi A. Jain A. Jain M. (2020). *Leptomonas seymouri* co-infection in cutaneous leishmaniasis cases caused by *Leishmania donovani* from Himachal Pradesh, India. Front. Cell Infect Microbiol. 10:345. doi: 10.3389/fcimb.2020.00345, PMID: 32760679 PMC7373763

[ref71] World Health Organization. (2023) Fact sheets: Leishmaniasis. Available at: https://www.who.int/news-room/fact-sheets/detail/leishmaniasis (Accessed June 1, 2023)

[ref72] Zhang X. Goncalves R. Mosser D. M. (2008). The isolation and characterization of murine macrophages. Curr. Protoc. Immunol. 14, 14.1.1–14.1.14. doi: 10.1002/0471142735.im1401s83, PMID: 19016445 PMC2834554

[ref73] Zídková L. Cepicka I. Votýpka J. Svobodová M. (2010). *Herpetomonas trimorpha* sp. nov. (Trypanosomatidae, Kinetoplastida), a parasite of the biting midge *Culicoides truncorum* (Ceratopogonidae, Diptera). Int. J. Syst. Evol. Microbiol. 60, 2236–2246. doi: 10.1099/ijs.0.014555-0, PMID: 19819998

