# Supplementary material for: Natural infection with Leishmania (Mundinia) martiniquensis supports Culicoides peregrinus (Diptera: Ceratopogonidae) as a potential vector of leishmaniasis and characterization of a Crithidia sp. isolated from the midges
Source: Front Microbiol. 2023 Aug 22;14:1235254. doi: 10.3389/fmicb.2023.1235254 (PMC10478001; doi:10.3389/fmicb.2023.1235254)
Supplement: Supplementary file 3 [file Data_Sheet_1.docx]

Supplementary Material

**Natural infection with *Leishmania* (*Mundinia*) *martiniquensis* supports *Culicoides peregrinus* (Diptera: Ceratopogonidae) as a potential vector of leishmaniasis and characterization of a *Crithidia* sp. isolated from the midges**

**Saowalak Kaewmee^1,2^, Chonlada Mano^3^, Thanari Phanitchakun^3^, Rinnara Ampol^2^, Thippawan Yasanga^4^, Urassaya Pattanawong^5^, Anuluck Junkum^3^, Padet Siriyasatien^2^, Paul A. Bates^6^, Narissara Jariyapan^2^***

*** Correspondence:** Narissara Jariyapan: narissara.j@chula.ac.th

**Supplementary Data 1.** PCR conditions and protocols used in this study.

All reactions were conducted using the Applied Biosystems Veriti (GenPlus®, USA). Each reaction mixture (25 μL) contained 6 μL of DNA template, 0.40 μL of each primer (10 μM), 0.5 μL of dNTPs (10 mM), 2.5 μL of 10X PCR buffer, 1.25 μL of MgCl_2_ (50 mM), 0.2 μL of Taq polymerase, and 13.75 μL of nuclease free water.

The protocol for *COI* gene [LCO1490 primer (5 ́- GGTCAACAAATCATAAAGATATTGG -3 ́) and HCO2198 primers (5 ́-TAAACTTCAGGGTGACCAAAAAATCA -3 ́)]: PCR amplification reaction was performed with an initial denaturation step at 94 °C for 5 min, followed by 5 cycles of the first amplification step (94 °C for 30 sec, 45 °C for 40 sec, and 72 °C for 1 min), then followed by 35 cycles of the second amplification step (94 °C for 30 sec, 51°C for 30 sec, and 72°C for 1 min), and followed by a final extension step at 72 °C for 10 min.

The protocol for *cyt b* gene [cyt bb1 primer (5 ́- CCATCMAACATYTCADCATGAAA -3 ́) and cyt bb2 primer (5 ́- GCHCCTCAGAATGAYATTTGKCCTCA -3 ́)]: PCR amplification reaction was performed with an initial denaturation step at 94 °C for 5 min, followed by 35 cycles of an amplification step (94 °C for 1 min, 55 °C for 1 min, and 72 °C for 1 min), and followed by a final extension step at 72 °C for 10 min.

The protocol for ITS1 region [LeF primer (5 ́- TCCGCCCGAAAGTTCACCGATA -3 ́) and LeR primer (5 ́- CCAAGTCATCCATCGCGACACG -3 ́)]: PCR amplification reaction was performed with an initial denaturation step at 95 °C for 5 min, followed by 40 cycles of an amplification step (95 °C for 1 min, 65 °C for 1 min, and 72 °C for 1 min), and followed by a final extension step at 72 °C for 10 min.

The protocol for *HSP70-I* gene [70-IR-D primer (5 ́- CCAAGGTCGAGGAGGTCGACTA-3 ́) and 70-IR-M primer (5 ́- ACGGGTAGGGGGAGGAAAGA -3 ́)]: PCR amplification reaction was performed with an initial denaturation step at 95 °C for 2 min, followed by 30 cycles of an amplification step (95 °C for 30 sec, 62.5 °C for 30 sec, and 72 °C for 1 min), and followed by a final extension step at 72 °C for 10 min.

The protocol for SSU rRNA gene [TRY927F primer (5 ́- GAAACAAGAAACACGGGAG -3 ́) and TRY927R primer (5 ́- CTACTGGGCAGCTTGGA -3 ́)]: PCR amplification reaction was performed with an initial denaturation step at 95 °C for 5 min, followed by 40 cycles of an amplification step (95 °C for 45 sec, 53 °C for 1 min, and 72 °C for 1 min 20 sec), and followed by a final extension step at 72 °C for 10 min.

The protocol for gGAPDH gene [M200 primer (5 ́- ATGGCTCCVVTCAARGTWGGMAT -3 ́) and M201 primer (5 ́- TAKCCCCACTCRTTRTCRTACCA - 3 ́)]: PCR amplification reaction was performed with an initial denaturation step at 94 °C for 3 min, followed by 40 cycles of an amplification step (94 °C for 30 sec, 50 °C for 30 sec, and 72 °C for 1 min 30 sec), and followed by a final extension step at 72 °C for 10 min.

**Supplementary Table 1.** Total number of *Culicoides* species captured in the two locations in Nakhon Si Thammarat, Thailand.

| **Species** | **Males** | **Females** | | | | **Total** |
| --- | --- | --- | --- | --- | --- | --- |
| **Tha Ruea** |  | **Parous** | **Engorged** | **Gravid** | **Nulliparous** |  |
| *C. peregrinus* | 5 | 266 (40)^a^ | 2 | 0 | 70 (49) | 343 (89) |
| *C. mahasarakhamense* | 1 | 8 (8) | 0 | 0 | 0 | 9 (8) |
| *C. oxystoma* | 0 | 2 (2) | 0 | 0 | 1 (1) | 3 (3) |
| Total |  | 276 (50) | 2 | 0 | 71(50) |  |
|  | 6 | 349 (100) | | | | 355 (100) |
|  |  |  | | | |  |
| **Khuan Phang** |  | **Parous** | **Engorged** | **Gravid** | **Nulliparous** |  |
| *C. peregrinus* | 15 | 517 (40) | 12 | 0 | 79 (40) | 623 (80) |
| *C. mahasarakhamense* | 0 | 1 (1) | 0 | 0 | 19 (2) | 20 (3) |
| *C. oxystoma* | 0 | 2 (2) | 0 | 0 | 0 | 2 (2) |
| *C. imicola* | 0 | 7 (7) | 0 | 0 | 24 (2) | 31 (9) |
| *C. shortti* | 0 | 0 | 0 | 0 | 27 (2) | 27 (2) |
| *C. huffi* | 0 | 0 | 0 | 0 | 25 (2) | 25 (2) |
| *C. palpifer* | 0 | 0 | 0 | 0 | 11 (2) | 11 (2) |
| Total |  | 527 (50) | 12 | 0 | 185 (50) |  |
|  | 15 | 724 (100) | | | | 739 (100) |

^a^Number of dissected insects

**Supplementary Table 2.** Data for growth curves of *Crithidia* sp. CLA-KP1 strain cultured in SIM complete for seven days at 26 °C and 37 °C. Results are expressed as mean ± standard deviation based on three independent replicates.

| Day | Number of parasites (×10^8^ cells/mL) | |
| --- | --- | --- |
|  | 26 ºC | 37 ºC |
| 0 | 0.0004±0.00 | 0.0004±0.00 |
| 1 | 0.03±0.01 | 0.04±0.00 |
| 2 | 0.18±0.04 | 0.37±0.07 |
| 3 | 0.53±0.07 | 0.64±0.09 |
| 4 | 1.20±0.34 | 0.98±0.01 |
| 5 | 1.49±0.17 | 0.96±0.01 |
| 6 | 1.90±0.24 | 0.71±0.10 |
| 7 | 1.48±0.10 | 0.01±0.00 |

**Supplementary Table 3.** Data for infection rate, average number of intracellular parasites per macrophage, infection index, and intracellular parasite multiplication ratio of *L. martiniquensis* (control) and *Crithidia* sp. CLA-KP1. Results are expressed as mean ± standard deviation based on three independent replicates.

| Time post infection (h) | Infection rate (%) | Average number of intracellular parasites/macrophage | Infection index | Intracellular parasite multiplication ratio |
| --- | --- | --- | --- | --- |
| *L. martiniquensis* (control) | | | | |
| 24 | 35.25±2.15 | 6.11±0.46 | 181.75±19.72 | 1.00±0.00 |
| 48 | 34.17±1.58 | 4.19±0.12 | 128.08±2.02 | 0.71±0.08 |
| 72 | 32.75±0.90 | 3.58±0.09 | 94.67±5.06 | 0.52±0.03 |
| 96 | 31.00±3.93 | 3.36±0.18 | 70.83±2.93 | 0.39±0.05 |
| *Crithidia* sp*.* | | | | |
| 24 | 22.27±0.55 | 2.35±0.09 | 51.97±0.45 | 1.00±0.00 |
| 48 | 6.68±1.85 | 1.56±0.17 | 10.25±2.10 | 0.21±0.04 |
| 72 | 0.00±0.00 | 0.00±0.00 | 0.00±0.00 | 0.00±0.00 |
| 96 | 0.00±0.00 | 0.00±0.00 | 0.00±0.00 | 0.00±0.00 |
